# BBSome deficiency in *Lotmaria passim* reveals divergent functions in trypanosomatid parasites

**DOI:** 10.1186/s13071-025-06704-3

**Published:** 2025-02-18

**Authors:** Xuye Yuan, Tatsuhiko Kadowaki

**Affiliations:** Department of Biosciences and Bioinformatics, Xi’an Jiaotong-Liverpool University, 111 Ren’ai Road, Suzhou Dushu Lake Higher Education Town, Suzhou, 215123 Jiangsu Province China

**Keywords:** BBSome, Trypanosomatid parasite, *Lotmaria passim*, Lipid raft

## Abstract

**Background:**

The BBSome is an octameric protein complex crucial for ciliary transport, though it also participates in multiple other cellular processes. These diverse functions may result from the co-option of its ancestral roles. Studying the BBSome in flagellated protists can provide insights into these ancestral functions and their subsequent adaptations.

**Methods:**

We examined the functions of the BBSome (LpBBS1 and LpBBS2) in *Lotmaria passim*, a monoxenous trypanosomatid parasite infecting honey bees. The phenotypes resulting from depletion of LpBBS1 using the auxin-inducible degron system and disruption of *LpBBS2* were characterized.

**Results:**

Parasites deficient in LpBBS2 are smaller and less motile compared with wild-type. Although intraflagellar transport of a marker membrane protein is only mildly impaired, its association with lipid rafts is significantly disrupted in the mutants. This suggests that the BBSome is essential for maintaining lipid raft integrity in *L. passim*. Transcriptomic comparisons between wild-type and *LpBBS2*-deficient parasites reveal that the BBSome may also influence processes related to metabolism, membrane localization of specific proteins, DNA repair, microtubules, and mitochondria.

**Conclusions:**

In contrast to *Leishmania mexicana*, the BBSome in *L. passim* is crucial for efficient infection of the honey bee gut, demonstrating that its cellular functions vary between related trypanosomatid species. The BBSome is likely an adaptor that links multiple proteins in a species-specific manner under various cellular contexts.

**Graphical Abstract:**

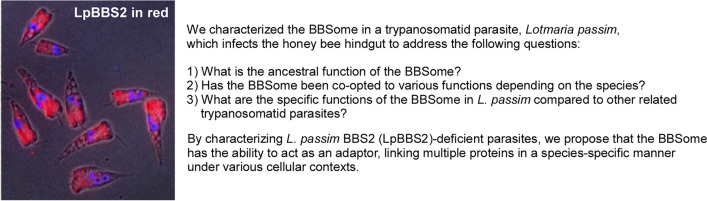

**Supplementary Information:**

The online version contains supplementary material available at 10.1186/s13071-025-06704-3.

## Background

The assembly of cilia-specific proteins requires three critical processes: the trafficking of proteins to the cilium, selective passage through the transition zone [1], and intraflagellar transport (IFT) [2]. The BBSome, an octameric complex of BBS1, BBS2, BBS4, BBS5, BBS7, BBS8, BBS9, and BBS18 [3–6], was originally suggested to function for ciliary transmembrane protein import [7, 8]. The BBSome is enriched at the transition zone [9] and moves bidirectionally during IFT associated with the IFT-A and IFT-B complexes [10]. Accumulation of the transmembrane proteins in cilium by the loss of BBSome led to the hypothesis that the BBSome promotes the export of ciliary transmembrane proteins [11, 12], while IFT-A stimulates their entry [13]. Indeed, the BBSome was involved in the exit of phospholipase D, SSTR3, and Smoothened from the cilium [14, 15]. In addition to the ciliary functions, the BBSome has several other intracellular functions, including regulating intracellular vesicular traffic [16], cell cytoskeleton dynamics [17], gene expression [18], proteasome-dependent protein degradation [19], and mitochondrial homeostasis [20]. Many phenotypes associated with BBSome deficiency could be explained by ciliary defects, but some may also result from the non-ciliary functions.

Despite extensive studies on the BBSome in mammals, there are few reports on its role in trypanosomatid parasites. *Leishmania major* lacking BBS1 shows no defects in growth, flagellar assembly, motility, or differentiation in vitro, but fails to establish infection in mice [21]. Similarly, in *Trypanosoma brucei*, the BBSome is not essential for flagellar morphogenesis, motility, general endocytosis, and cell viability, but it is necessary for virulence. These findings suggest that the BBSome facilitates endocytic sorting of specific membrane proteins at the ciliary base through interactions with clathrin and ubiquitin [22]. However, its role in ciliary transport in these parasites remains unclear.

Given the conservation of BBSome subunits in flagellated protists, studying its function in different organisms can provide insight into its ancestral roles. In this study, we focus on *Lotmaria passim*, a monoxenous trypanosomatid that infects honey bee hindguts globally [23]. This parasite negatively impacts honey bee health [24]. Our investigation reveals that LpBBS1 is essential for viability. To further study its function, we tested the applicability of the auxin-inducible degron (AID) system [25, 26] in *L. passim*. The AID system is a tool to control protein degradation in various model organisms, including yeast, mammalian cells, and plants. It is possible to rapidly and reversibly degrade specific proteins of interest by tagging them with a degron sequence derived from the plant protein IAA7. When the plant hormone auxin (indole-3-acetic acid, IAA) is added to the system, it promotes interaction between the degron-tagged protein and an engineered Skp1-Cullin-F-box (SCF) ubiquitin ligase complex containing a plant F-box protein, AFB2. This interaction leads to the ubiquitination and subsequent proteasomal degradation of the tagged protein. Meanwhile, we found that LpBBS2 is necessary for proper morphogenesis, motility, flagellar transport, lipid raft integrity, and successful honey bee infection. Diverse functions of the BBSome for various cellular processes in flagellated protists will be discussed.

## Methods

### Culture of *L. passim*

*L. passim* promastigotes (ATCC PRA-403) were maintained in a modified FP-FB medium [27] (Additional file 1, Supplementary Dataset 1) at 21 °C.

### Cellular localization of 3c-Myc-LpBBS1, 3c-Myc-LpBBS2, and LpIFT88-green fluorescent protein (GFP) in *L. passim*

To express the triple c-Myc-tagged proteins 3c-Myc-LpBBS1 and 3c-Myc-LpBBS2, two oligonucleotides, 3Myc-N-5 and 3Myc-N-3, were phosphorylated and annealed, and then cloned into the XbaI and HindIII sites of the tdTomato/pTREX-b plasmid DNA [28] (ADDGENE: #68709). The full open reading frames (ORFs) of the *LpBBS1* and *LpBBS2* genes were amplified using polymerase chain reaction (PCR) with KOD-FX DNA polymerase (TOYOBO), *L. passim* genomic DNA, and primer pairs LpBBS1-5-HindIII/LpBBS1-3-ClaI and LpBBS2-5-ClaI/LpBBS2-3-ClaI, respectively. PCR products were digested with HindIII and ClaI for *LpBBS1* and with ClaI for *LpBBS2*. The digested products were cloned into the vector mentioned above, which had been digested with the same enzymes. To construct the LpIFT88-GFP expression vector, the ORF of *LpIFT88* was amplified using primers LpIFT88-5-XbaI and LpIFT88-3-XbaI. The PCR product was digested with XbaI and cloned into the XbaI site of the pTrex-*n*-eGFP plasmid (ADDGENE: #62544). *LpBBS1*, *LpBBS2*, and *LpIFT88* sequences are listed in Additional file 2: Supplementary Dataset 2.

Actively growing *L. passim* cells (4 × 10^7^) were washed twice with 5 mL phosphate-buffered saline (PBS) and resuspended in 0.4 mL Cytomix buffer (without EDTA) containing 20 mM KCl, 0.15 mM CaCl_2_, 10 mM K_2_HPO_4_, 25 mM HEPES, and 5 mM MgCl_2_ (pH 7.6). Electroporation was performed twice (1-min interval) using 10 μg of each plasmid DNA and a Gene Pulser X cell electroporator (Bio-Rad) with a 2-mm gap cuvette, setting the voltage to 1.5 kV, capacitance to 25 μF, and resistance to infinity. The electroporated parasites were cultured in 4 mL of modified FPFB medium, and blasticidin (50 μg/mL, Macklin) or G418 (200 μg/mL, Sigma-Aldrich) was added after 24 h for selection of drug-resistant clones.

Immunofluorescence detection of 3c-Myc-LpBBS1 and 3c-Myc-LpBBS2 was performed by washing and mounting the parasites on a poly-l-lysine-coated 8-well chamber slide, fixing with 4% paraformaldehyde, permeabilizing with 0.1% Triton X-100 in PBS (PT), and blocking with PT-containing 5% normal goat serum (PTG). Samples were incubated overnight at 4 °C with a rabbit anti-c-Myc polyclonal antibody (1:500 dilution, Proteintech) in PTG. After five washes with PT, the samples were incubated with Alexa Fluor 555 anti-rabbit immunoglobulin IgG (ThermoFisher) for 2 h at room temperature, washed again, stained with 4′,6-diamidino-2-phenylindole (DAPI) (1 µg/mL) for 5 min, and briefly washed with PBS. The samples were observed under a NIKON Eclipse Ni-U fluorescence microscope with a constant exposure time of 200 ms. Live *L. passim* expressing LpIFT88-GFP were washed three times with 1 mL PBS and imaged on poly-l-lysine-coated slides.

### Testing the AID System Using GFP in *L. passim*

To tag green fluorescent protein (GFP) with a degron, the miniIAA7 sequence was amplified using primers miniIAA-5-XbaI and miniIAA-3-XbaI, as well as pSH-EFIRES-B-Serpin-miniIAA7-mEGFP plasmid DNA [26] (ADDGENE: # 129719) as a template, and cloned into the pTrex-*n*-eGFP plasmid. The complete ORF of *AtAFB2* was amplified using primers AtAFB2-5-XbaI and AtAFB2-3-HindIII, as well as pSH-EFIRES-P-AtAFB2 plasmid DNA [26] (ADDGENE: #129715) as a template, and cloned into the tdTomato/pTREX-b plasmid. *L. passim* cells were electroporated with both plasmids (10 μg each), and drug-resistant clones were selected using G418 and blasticidin.

The parasites expressing miniIAA7-GFP and AtAFB2 were cultured inoculated at 10^6^/mL in 24-well plates with 75 μg/mL auxin (IAA, Macklin) for 0–24 h at 30 °C. Cells were collected, washed with 1 mL PBS, and lysed in 100 μL sodium dodecyl-sulfate polyacrylamide gel electrophoresis (SDS-PAGE) sample buffer (2% SDS, 10% glycerol, 10% β-mercaptoethanol, 0.25% bromophenol blue, 50 mM Tris–HCl, pH 6.8). Samples were heated at 95 °C for 5 min, and 20 μL was applied to two 10% SDS-PAGE gels. One gel was stained with Instant Blue (Abcam), while proteins from the other were transferred to a nitrocellulose membrane (Pall Life Sciences). The membrane was blocked with 5% BSA in PBS with 0.1% Tween-20 (PBST) and incubated with anti-GFP polyclonal antibody (1:500 dilution, Proteintech) overnight at 4 ℃. After washing five times with PBST (for 5 min each), the membrane was incubated with IRDye 680RD donkey anti-rabbit IgG (H + L) secondary antibody (1:10,000 dilution, LI-COR Biosciences) in PBST containing 5% skim milk at room temperature for 2 h. After another round of washing, the membrane was visualized using ChemiDoc MP (BioRad).

### Tagging endogenous *LpBBS1* with triple c-Myc epitopes and miniIAA7 by clustered regularly interspaced short palindromic repeats (CRISPR)

To tag the *LpBBS1* gene, the guide RNA (gRNA) sequence targeted at 29 bp downstream of the stop codon of *LpBBS1* was designed using a custom gRNA design tool (http://grna.ctegd.uga.edu) [29]. Complementary oligonucleotides corresponding to the sgRNA sequences (LpBBS1gRNA3′UTR48F and LpBBS1gRNA3′UTR48R) were phosphorylated by T4 polynucleotide kinase (TAKARA), followed by annealing and cloning into BbsI-digested pSPneogRNAH vector [30] (ADDGENE: # 63556). *L. passim* expressing Cas9 [31] was electroporated with 10 μg of the constructed plasmid DNA and the parasites expressing both Cas9 and *LpBBS1* gRNA were selected by blasticidin and G418.

For the construction of donor DNA to tag the *LpBBS1* gene, the following two PCR products were fused: the *T. cruzi Gapdh* terminator sequence derived from pTrex-*n*-eGFP (GAPDH-HindIII and GAPDH-3-Hyg) and the ORF of the *Hph* gene derived from pCsV1300 [32] (Hyg-5-GAPDH and Hyg-3-EcoRI). The fusion PCR products were digested and cloned into the HindIII and EcoRI sites of pBluescript II SK( +). The truncated 3′UTR of *LpBBS1* was PCR-amplified using two primers, LpBBS1-3′UTR-F-degron and LpBBS1-3′UTR-R. The PCR product was digested and cloned into the EcoRI site of the aforementioned plasmid DNA. The full-length *LpBBS1* tagged with triple c-Myc epitopes and miniIAA7 was constructed as follows. The PCR amplicon of miniIAA7, using two primers (miniIAA7-5-HindIII and miniIAA7-3-XhoI), was digested and cloned into the HindIII and XhoI sites of pBluescript II SK( +). The DNA fragment encoding triple c-Myc epitopes was then cloned into the XbaI and HindIII sites of the aforementioned plasmid DNA. By cutting the resulting plasmid DNA with XbaI and XhoI, the DNA fragment encoding triple c-Myc epitopes and miniIAA7 was obtained. The complete ORF of *LpBBS1*, amplified by two primers (LpBBS1-5-XbaI and LpBBS1-3-XbaI), along with the aforementioned DNA fragment, was cloned into the XbaI and XhoI sites of pTrex-*n*-eGFP. Using this plasmid DNA as a template, a DNA fragment containing the *LpBBS1* ORF (703–1776), triple c-Myc epitopes, and miniIAA7 was amplified using two primers, LpBBS1-5-degron and miniIAA7-3-ClaI. The PCR product was digested and cloned into the XhoI and ClaI sites of pBluescript II SK( +) containing the *T. cruzi Gapdh* terminator, *Hph*, and truncated 3′UTR of *LpBBS1* as described above. The donor plasmid DNA (10 μg) was linearized using NotI and used for electroporation of *L. passim* expressing both Cas9 and *LpBBS1* gRNA.

After electroporation, *L. passim* resistant to blasticidin, G418, and hygromycin (150 μg/mL, Macklin) was selected and cloned by serial dilutions in a 96-well plate. The genotype of each clone was determined by the detection of 3′ wild-type (WT, using LpBBS1-1681F and LpBBS1-3′UTR-down) and tagged (T, using Hyg-846F and LpBBS1-3′UTR-down) alleles for *LpBBS1* by PCR. The reverse primer, LpBBS1-3′UTR-down, which corresponds to the *LpBBS1* 3′UTR sequence, was used for this diagnostic PCR. To detect the wild-type allele, the forward primer LpBBS1-1681F, targeting the *LpBBS1 ORF* sequence, was used to produce an 827 bp amplicon. To detect the tagged allele, the forward primer Hyg-846F, targeting the *Hph* ORF sequence, was used to produce a 909 bp amplicon (Fig. 2B). For both wild-type and tagged allele detection, the PCR extension time was set to 40 s. This ensured that a 2.9 kb amplicon (comprising 3′end of *LpBBS1* ORF, 3c-Myc epitopes, miniIAA7, *T. cruzi Gapdh* terminator, *Hph*, and the part of *LpBBS1* 3′UTR sequences) was not amplified in homozygous tagged clones (Fig. 2C). The PCR for detecting tagged alleles was performed using a two-step cycling method to minimize nonspecific band. The *LpBBS1*-tagged *L. passim* (LpBBS1-3c-Myc-miniIAA7) was immunostained using an anti-c-Myc polyclonal antibody, following the same procedure as described above. Two independent clones (D1 and E9) were electroporated with the *AtAFB2*-expressing vector, in which the blasticidin resistance gene was replaced by the bleomycin resistance gene. LpBBS1-3c-Myc-miniIAA7 parasites expressing *AtAFB2* were selected by hygromycin and zeocin (50 μg/mL, InvivoGen).

### AID-mediated degradation of *LpBBS1* and its effect on the growth rate of *L. passim*

LpBBS1-3c-Myc-miniIAA7 parasites expressing AtAFB2 were cultured with indole-3-acetic acid (IAA) for 5 and 10 days at 30 °C. Then, 10^6^ cells were collected, along with untreated and WT parasites, during the mid-log growth phase. Cell lysates were prepared as described above and applied to 8% SDS-PAGE. Rabbit anti-c-Myc (1:1000 dilution) polyclonal antibody was used for the western blot. WT and LpBBS1-3c-Myc-miniIAA7 parasites expressing AtAFB2 were inoculated into the culture medium at 10^4^ cells/mL in a 24-well plate and cultured with or without IAA at 30 °C. The number of parasites was counted daily using a hemocytometer for 4 days. The phenotypes of the D1 and E9 clones were the same.

### Disruption of *LpBBS2* gene by CRISPR

To disrupt the *LpBBS2* gene, complementary oligonucleotides corresponding to the sgRNA sequences (LpBBS2gRNA1091F and LpBBS2gRNA1091R) were processed and cloned into the pSPneogRNAH vector, as described above. *L. passim* expressing both Cas9 and *LpBBS2* gRNA was then established.

For the construction of donor DNA for the *LpBBS2* gene, three DNA fragments: the 5′UTR of *LpBBS2* (LpBBS2 5′UTR-F and LpBBS2 5′UTR-R), the ORF of the *Hph* gene (LpBBS2 Hph-F and LpBBS2 Hph-R), and part of the *LpBBS2* ORF (1271–1870, LpBBS2 3′ORF-F, and LpBBS2 3′ORF-R) were fused. The fusion PCR product was cloned into the EcoRV site of pBluescript II SK( +), and then the linearized plasmid DNA (10 μg) with XhoI was used for electroporation of *L. passim* expressing both Cas9 and *LpBBS2* gRNA, as described above.

After electroporation, *L. passim* resistant to blasticidin, G418, and hygromycin was selected and cloned by serial dilutions in a 96-well plate. The genotype of each clone was first determined by the detection of 5′ wild-type (WT) and knock-out (KO) alleles for *LpBBS2* by PCR. After identifying heterozygous ( ±) and homozygous (−/−) KO clones, their 5′WT (LpBBS2 5′UTR-Up and LpBBS2-47R), 5′KO (LpBBS2 5′UTR-Up and Hyg-159R), 3′WT (LpBBS2-1216F and LpBBS2-1910R), and 3′KO (Hyg-846F and LpBBS2-1910R) alleles were confirmed by PCR.

### RT-PCR

Total RNA from WT, *LpBBS2* heterozygous, and homozygous mutant parasites were extracted using TRIzol reagent (Sigma-Aldrich). Reverse transcription of total RNA (0.2 μg) was carried out using ReverTra Ace (TOYOBO) and random primers, followed by PCR with GoTaq Green Master Mix (Promega). *LpBBS2* mRNA was detected by nested PCR by running the first PCR with LpSL-F-1st and LpBBS2-70R primers, followed by the second PCR with LpSL-F-2nd and LpBBS2-47R primers. To detect *LpGAPDH* mRNA, LpSL-F and LpGAPDH-R primers were used. The LpSL-F primer matches the full-length splice leader sequence, while the LpSL-F-1st and LpSL-F-2nd primers correspond to partial sequences.

### Culture, flagellar and cell body length measurement, and motility measurement of *L. passim*

To construct plasmid DNA expressing *LpBBS2,* the ORF was first PCR amplified using LpBBS2-5-XbaI and LpBBS2-3-ClaI primers. The plasmid DNA to express 3c-Myc-LpBBS2 was used as a template. The resulting PCR product was digested with XbaI and ClaI followed by subcloning into the same restriction enzyme sites of AtAFB2-expressing vector with the bleomycin resistance gene. *LpBBS2*-deficient parasites were electroporated with above plasmid DNA and the parasites expressing *LpBBS2* (*LpBBS2*-rescued) were selected using hygromycin and zeocin.

For culture, flagellar and cell body length measurement, and motility measurement, WT, *LpBBS2*-deficient, and *LpBBS2*-rescued parasites were inoculated into the culture medium at 10^4^ cells/mL at 30 °C. The number of parasites was counted using a hemocytometer, and images of the cultured parasites were taken every day for 5 days. The length of both the flagellum and the cell body of individual parasites was measured at 3 days after culture using phase images and Image-J. To track the movement of parasites, videos were created by capturing images every second for 1 min. The movement of parasites was analyzed using TrackMate v7.10.2 [33] as a plugin in Fiji [34]. The videos were imported into Fiji, converted to 8-bit grayscale, and the brightness and contrast were adjusted to enhance tracking accuracy. The Laplacian of Gaussian detector was utilized with an estimated object diameter of 30.0–36.0 pixels and a quality threshold of 0.2–0.5 for the identification of individual parasites. The Simple Linear Assignment Problem tracker was employed to track parasites with adjustments made to the linking maximum distance and gap-closing maximum distance set to 35.0–200.0 pixels, and the gap-closing maximum frame gap set to 1. To examine the growth rates of WT, *LpBBS2*-deficient, and *LpBBS2*-rescued parasites at 21 °C, the cells were inoculated at 10^5^ cells/mL. The phenotypes of two independent *LpBBS2*-deficient clones (D8 and G1) were characterized, and their phenotypes were the same. Therefore, the D8 clone was used for further experiments.

### Honey bee infection

For infection of honey bees with *L. passim*, parasites were collected during the logarithmic growth phase (5 × 10^5^ cells/mL), washed with PBS and suspended in sterile 10% sucrose/PBS at 5 × 10^4^ cells/μL. Newly emerged honey bees were sampled by placing frames with late pupae in a 33 °C incubator and then starved for 2–3 h. A total of 20 individual honey bees were fed with 2 μL of the sucrose/PBS solution containing either WT, *LpBBS2*-deficient, or *LpBBS2*-rescued parasites (10^5^ cells in total). The infected honey bees were maintained in metal cages at 33 °C for 14 days and then frozen at −80 °C. This experiment was repeated three times. In total, 8 honey bees were sampled from each of the three experiments; thus 24 honey bees were analyzed in total, infected with either WT, *LpBBS2*-deficient, or *LpBBS2*-rescued parasites. Genomic DNA from the whole abdomens of individual bees was extracted using DNAzol reagent. *L. passim* was quantified in the infected honey bees by qPCR using LpITS2-F and LpITS2-R primers, that correspond to part of the internal transcribed spacer region 2 (ITS2) in the ribosomal RNA gene. Honey bee *AmHsTRPA* was used as the reference gene with AmHsTRPA-F and AmHsTRPA-R primers [24]. The relative abundance of *L. passim* in individual honey bees (24 each infected by WT or mutant *L. passim*) was calculated using the ΔCt method, setting one sample infected by WT as 1. Statistical analysis was conducted using the Brunner–Munzel test.

### Localization of FCaBP1N16::GFP and FCaBP2N16::GFP and their association with lipid rafts in WT and *LpBBS2*-deficient *L. passim*

Plasmid DNA encoding either FCaBP1N16::GFP or FCaBP2N16::GFP was digested with BamHI and XhoI, and then it was subcloned into the same sites of pTrex-*n*-eGFP plasmid DNA, wherein the neomycin resistance gene was replaced by the bleomycin resistance gene. WT and *LpBBS2*-deficient *L. passim* were electroporated with the plasmid DNA and selected by either zeocin (for WT) or hygromycin and zeocin (for *LpBBS2* mutants). Electroporation was also conducted using pTrex-*n*-eGFP plasmid DNA with the bleomycin resistance gene to express GFP. The GFP and DIC images of parasites were captured as described above. Image-J was used to measure the mean fluorescence of FCaBP1N16::GFP in both the entire flagellum and cell body of individual parasites, as well as the background. Subsequently, the ratio between the fluorescence in the flagellum and the cell body was calculated by subtracting the background fluorescence. Statistical comparison was conducted using the Brunner–Munzel test.

The association of FCaBP1N16::GFP and FCaBP2N16::GFP with lipid rafts in WT and *LpBBS2*-mutant parasites was tested along with GFP as a control. The cells in the mid-log growth phase were collected, washed twice in 0.5 mL PBS, and resuspended at 10⁷ cells/mL in 150 μL of 1% Triton X-100 in PBS, pre-equilibrated to either 4, 20, or 37 °C. The cells were incubated at the same temperature for 10 min and centrifuged at 1000*g* for 5 min. The supernatants were removed from the pellets and transferred to new tubes, and the pellets (insoluble fraction) were resuspended in 150 μL of 1% Triton X-100 in PBS. The supernatants were centrifuged again at 17,000*g* for 10 min at either 4, 20, or 37 °C, and the supernatants (soluble fraction) were collected. Both fractions were mixed with 50 μL of 4× SDS-PAGE sample buffer and heated at 95 °C for 5 min, and 35 μL of each sample was applied to 12% SDS-PAGE. The proteins were detected by western blot using anti-GFP antibody as described above. Image-J was used to measure the band intensities of FCaBP1N16::GFP and FCaBP2N16::GFP in the pellet and soluble fractions after background subtraction. Then, the ratio of pellet to soluble fraction was calculated and Welch’s *t*-test was used for statistical comparison.

### Transcriptome analysis of WT and *LpBBS2*-deficient *L. passim*

WT and *LpBBS2*-deficient parasites (clones D8 and G1) were cultured in three 10 cm culture plates for each genotype at 30 °C, and collected during the mid-log growth phase. Total RNA was independently extracted from each plate and analyzed by RNA-seq. All samples were sequenced on the DNBSEQ-T7 platform at the Beijing Genomics Institute, yielding at least 6 GB of clean data per sample.

Since the genome sequence of *L. passim* (strain 422, GenBank assembly: GCA_034478905.1) in NCBI lacks gene annotation information, *L. passim* protein-coding genes were first annotated. Gene annotation for the *L. passim* genome sequence was conducted using the BRAKER pipeline [35] with *Crithidia bombi*, *Leptomonas pyrrhocoris*, and *Leptomonas seymouri* as reference organisms. The gffread program (http://ccb.jhu.edu/software/stringtie/gff.shtml) was used to convert the *L. passim* GFF to a GTF file, and the genomic indices from the converted GTF file was generated using genomeGenerate from STAR (Version: 2.7.0) [36].

The quality of all RNA-seq data was analyzed using FastQC [37] and pruned using SOAPnuke (version 2.2.1). After trimming low-quality reads and removing reads with more than 5% adapter and N bases, more than 92% of the reads was retained in each sample for analysis. The clean RNA-seq reads were then mapped to the STAR index. The assembled reads were aligned to the *L. passim* genome using alignReads in STAR, and then coordinate-sorted BAM files were generated. The mapping rates of the nine samples to *L. passim* genome ranged from 73.05% to 73.78%. Principal component analysis (PCA) of the nine RNA-seq samples was conducted using the gmodels package (version 2.18.1) in R. As a preprocessing step for differential gene expression analysis, htseq-count [38] developed with HTSeq was used to count the overlap of reads mapped to the GFF features. Raw read counts were analyzed in RStudio (Version 1.2.1335) using the linear contrast function in the DESeq2 package (Version 1.24.0) from Bioconductor [39]. Normalization was conducted using shrinkage estimation in DESeq2 [40] and reads with fewer than one count per million mapped reads were removed from the analysis. The Benjamini–Hochberg method was applied to calculate the false discovery rate (FDR) and log2 fold change (FC) across the detected genes. Differentially expressed genes (DEGs) between two samples (WT versus D8, WT versus G1, and D8 versus G1) were identified using a threshold of FDR < 0.05 and log2 FC > 1. For scatter plot analysis, the logarithmic values of gene expression levels of two samples are plotted on the x- and y-axis, respectively.

GO enrichment analysis of DEGs was carried out using the clusterProfiler package (version 3.18.0) [41], and all statistical analyses were performed in RStudio (Version 1.2.1335). After obtaining the enriched GO terms, the most specific ones were identified using a cutoff value of FDR < 0.05. The accession number for the RNA-seq data is PRJNA1169641.

### qRT-PCR

WT and *LpBBS2*-deficient parasites were cultured in five 6 cm plates per genotype at 30 °C and harvested during the mid-log growth phase. Total RNA was extracted independently from each plate, and reverse transcription was performed in a 20 μL reaction volume, as previously described. The resulting cDNA was diluted twofold with water, and specific mRNA levels were quantified by qPCR using the primers LpSL-F and gene-specific reverse primers. *L. passim* 18S rRNA served as the reference gene, with the primers L. passim 18S-F and L. passim 18S-R [24]. The relative mRNA abundance was calculated using the ΔCt method, with one WT sample set as 1. Statistical analysis was performed using Welch’s *t*-test. A complete list of primers used can be found in Additional File 3: Supplementary Dataset 3.

## Results

### Intracellular localization of LpBBS1 and LpBBS2

**Fig. 1:**
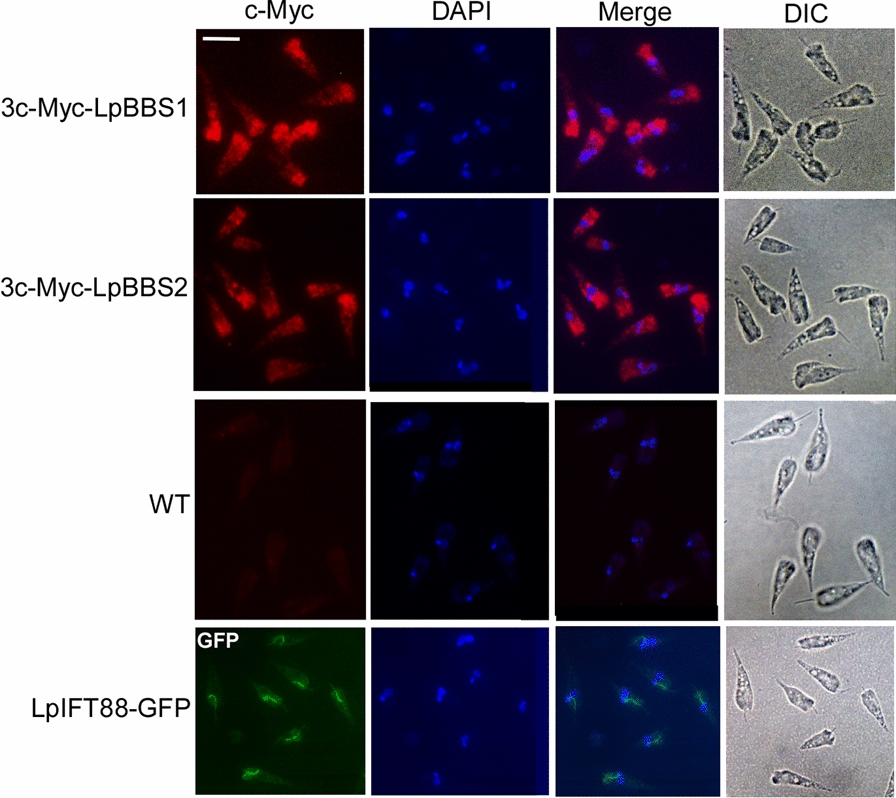
Cellular localizations of LpBBS1, LpBBS2, and LpIFT88-GFP. *Lotmaria passim* expressing 3c-Myc-LpBBS1 or 3c-Myc-LpBBS2, along with wild-type (WT) controls, were analyzed via immunofluorescence (c-Myc), DAPI staining, and differential interference contrast (DIC) microscopy. LpIFT88-GFP was directly visualized using fluorescence light (GFP). Merged images of c-Myc and DAPI are provided. Scale bar: 5 μm

To explore the role of the BBSome complex in *L. passim*, we examined the intracellular localization of LpBBS1 and LpBBS2 by tagging them with triple c-Myc epitopes and expressing them using episomal vectors. As shown in Fig. 1, both LpBBS1 and LpBBS2 are distributed throughout the cell body, with significant enrichment at the anterior end. This localization was absent in wild-type *L. passim*. In contrast, a subunit of IFT-B complex, LpIFT88-GFP was confined to the base of the flagellum. Previous studies have shown that the BBSome localizes to the flagellar pocket membrane in *T. brucei* and that BBS1 is present throughout the cell body in *L. major* [21, 22]. These results suggest that the BBSome likely performs multiple roles in *L. passim*.

### Application of auxin-inducible degron (AID) system to study LpBBS1 function

**Fig. 2:**
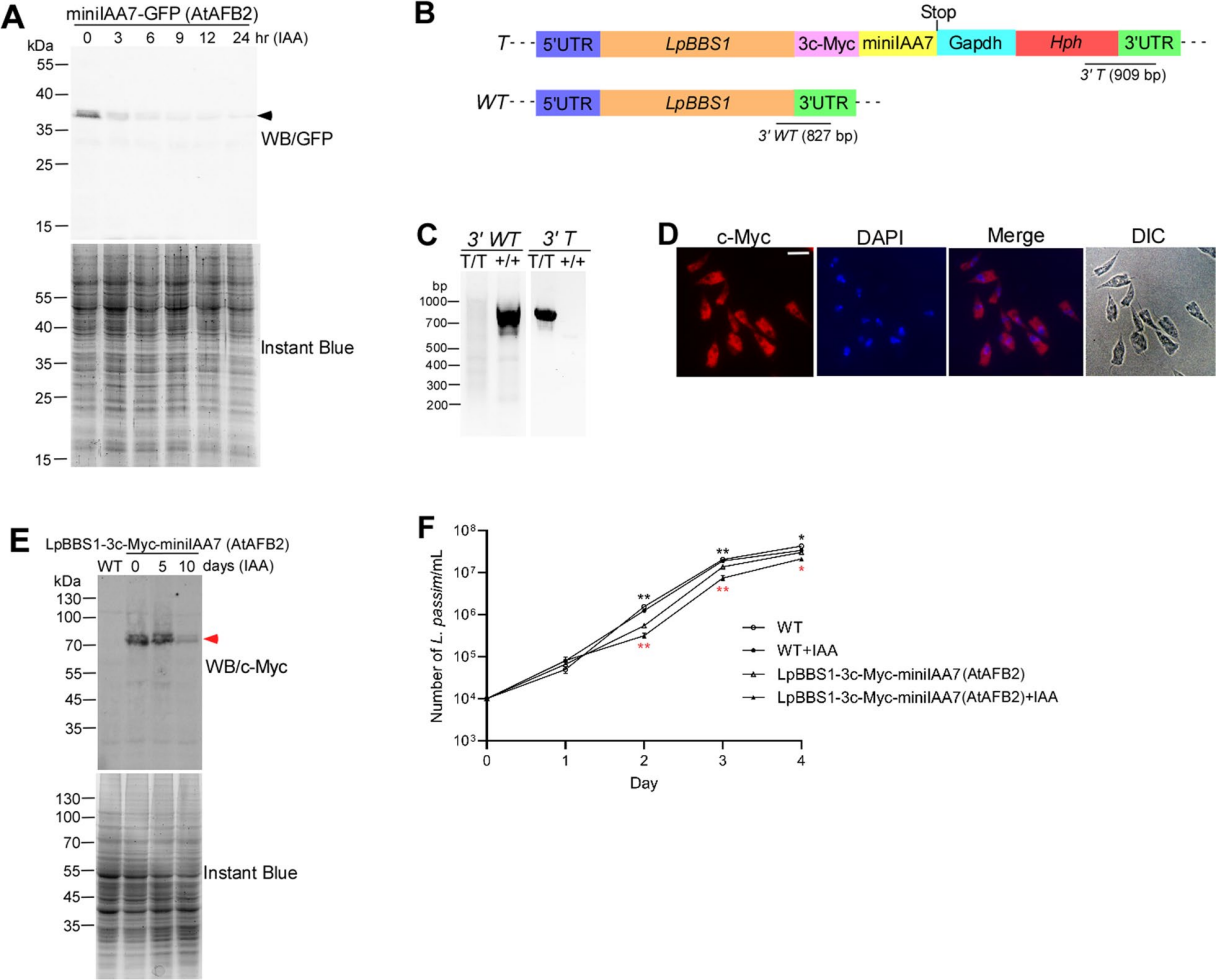
AID-mediated functional analysis of LpBBS1. **A** *L. passim* expressing degron-tagged GFP and AtAFB2, miniIAA7-GFP (AtAFB2), was treated with IAA for 0–24 h. Cell lysates were analyzed by 12% SDS-PAGE, followed by western blot with anti-GFP antibody (WB/GFP) and Instant Blue staining. The miniIAA7-GFP band is marked by an arrowhead. Protein marker molecular weights (kDa) are indicated on the left. **B** Schematic representation of wild-type (*WT*) and tagged (*T*) alleles of *LpBBS1*, created via CRISPR/Cas9-induced homology-directed repair. The 5′ and 3′ untranslated regions (UTRs), open reading frame (ORF), 3c-Myc epitopes, miniIAA7, *Trypanosoma cruzi Gapdh* terminator, and hygromycin resistance gene (*Hph*) are color-coded. The stop codon is indicated. Expected PCR product sizes for detecting *3′ WT* and *3′ T* alleles (not to scale) are also shown. **C** Genomic DNA from WT (+ / +) and homozygous tagged *LpBBS1* clones (T/T) was analyzed by PCR to detect 3′ *WT* and 3′ *T* alleles. Molecular weight marker sizes are shown on the left. **D** The homozygous tagged *LpBBS1* clone, LpBBS1-3c-Myc-miniIAA7, was examined using immunofluorescence (c-Myc), DAPI staining, and differential interference contrast (DIC) microscopy. The merged images of c-Myc and DAPI are also shown. Scale bar: 5 μm. **E** Cell lysates from LpBBS1-3c-Myc-miniIAA7 parasites expressing AtAFB2, LpBBS1-3c-Myc-miniIAA7 (AtAFB2), treated with IAA for 0–10 days, and untreated WT parasites were analyzed by 8% SDS-PAGE. LpBBS1-3c-Myc-miniIAA7 protein (red arrowhead) was detected via western blot using anti-c-Myc antibody (WB/c-Myc), with total proteins visualized by Instant Blue staining. **F** Growth rates of WT (white circles), WT with IAA (+ IAA, black circles), LpBBS1-3c-Myc-miniIAA7 (AtAFB2) (clone E9, white triangles), and LpBBS1-3c-Myc-miniIAA7 (AtAFB2) with IAA (+ IAA, black triangles) were monitored in culture for 4 days at 30 °C (biological replicates, *n* = 3). Growth differences between WT and LpBBS1-3c-Myc-miniIAA7 (AtAFB2) without IAA (black asterisks, day 2; *P* < 0.002, day 3; *P* < 0.003, day 4; *P* < 0.02) and between treated and untreated LpBBS1-3c-Myc-miniIAA7 (AtAFB2) (red asterisks, day 2; *P* < 0.003, day 3; *P* < 0.002, day 4; *P* < 0.02) were statistically significant (two-tailed Welch’s *t*-test)

We attempted to disrupt the *LpBBS1* gene using CRISPR-based homology-directed repair (HDR), as previously reported [31]. However, we were unable to generate homozygous knock-out clones, even with two gRNAs targeting different sites within the *LpBBS1* ORF. Thus, LpBBS1 is likely to be essential for *L. passim* viability. To further study its function, we applied the AID system in *L. passim*. We created parasites expressing a miniIAA7-tagged GFP and *Arabidopsis thaliana* AFB2 (AtAFB2) on episomal vectors. Upon the addition of auxin (IAA), the miniIAA7-tagged GFP was rapidly degraded (Fig. 2A), confirming the functionality of the AID system in *L. passim*.

To tag the endogenous LpBBS1 with triple c-Myc epitopes and miniIAA7, we constructed a HDR plasmid DNA made with 3′ORF of *LpBBS1*, triple c-Myc epitopes, miniIAA7, *Trypanosoma cruzi Gapdh* terminator sequence derived from pTrex-*n*-eGFP [42], hygromycin-resistant gene (*Hph*), and truncated 3′UTR of *LpBBS1* (Fig. 2B). This construct was electroporated to the parasites expressing Cas9 and gRNA targeted at 29 bp downstream of the stop codon of *LpBBS1*. Successful integration of this construct into the parasite genome was confirmed by PCR (Fig. 2C), creating homozygous tagged clones (LpBBS1-3c-Myc-miniIAA7). The tagged endogenous LpBBS1 exhibited the same localization pattern as the ectopic LpBBS1 (Figs. 1 and 2D), indicating that this is the authentic intracellular localization. Western blot analysis confirmed the expression of the tagged LpBBS1. However, depletion of the tagged LpBBS1 upon IAA treatment was slow, requiring more than 5 days, and the protein remained detectable even after 10 days (Fig. 2E). Indeed, we were able to maintain the LpBBS1-3c-Myc-miniIAA7 clone expressing AtAFB2 in the continuous presence of IAA, but its growth was slower compared with the untreated one. Moreover, the LpBBS1-3c-Myc-miniIAA7 clone expressing AtAFB2 proliferated more slowly than the wild type, even without IAA, at 30 ℃ (Fig. 2F). These results indicate that LpBBS1 is crucial for normal growth in *L. passim* and that tagging with triple c-Myc epitopes and miniIAA7 impairs its function under culture conditions. Disruption of *BBS1* genes in *L. major* and *T. brucei* did not affect the normal growth [21, 22], suggesting that LpBBS1 may carry out the functions that are absent in the above trypanosomatid parasites.

### Role of LpBBS2 in proliferation, morphology, and motility

**Fig. 3:**
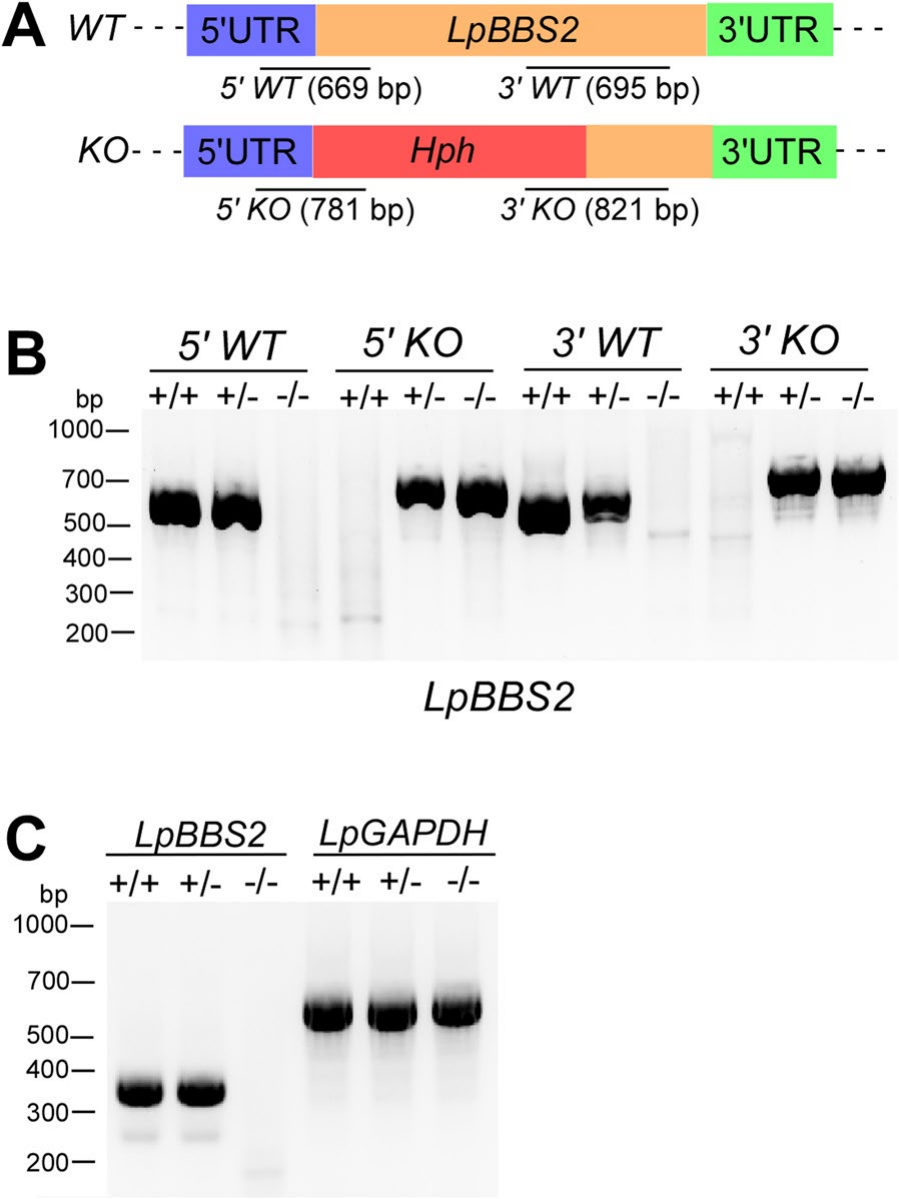
CRISPR-mediated disruption of *LpBBS2.* **A** Schematic representation of *WT* and disrupted (*KO*) alleles of *LpBBS2* generated via CRISPR/Cas9-induced homology-directed repair. 5′ and 3′ UTRs, ORF, and *Hph* are color-coded. Expected PCR product sizes for detecting 5′ *WT, 3′ WT, 5′ KO,* and 3′ *KO* alleles (not to scale) are shown. **B** Genomic DNA from WT (+ / +), heterozygous ( ±), and homozygous (−/−) *LpBBS2*-mutant parasites was analyzed by PCR for *5′ WT*, *5′ KO*, *3′ WT*, and *3′ KO* alleles. Molecular weight markers are shown on the left. **C** RT-PCR detection of *LpBBS2* and *LpGAPDH* mRNAs in *LpBBS2* heterozygous and homozygous mutants, along with WT parasites, using a forward primer targeting the *L. passim* splice leader sequence. Molecular weight markers are shown on the left

**Fig. 4:**
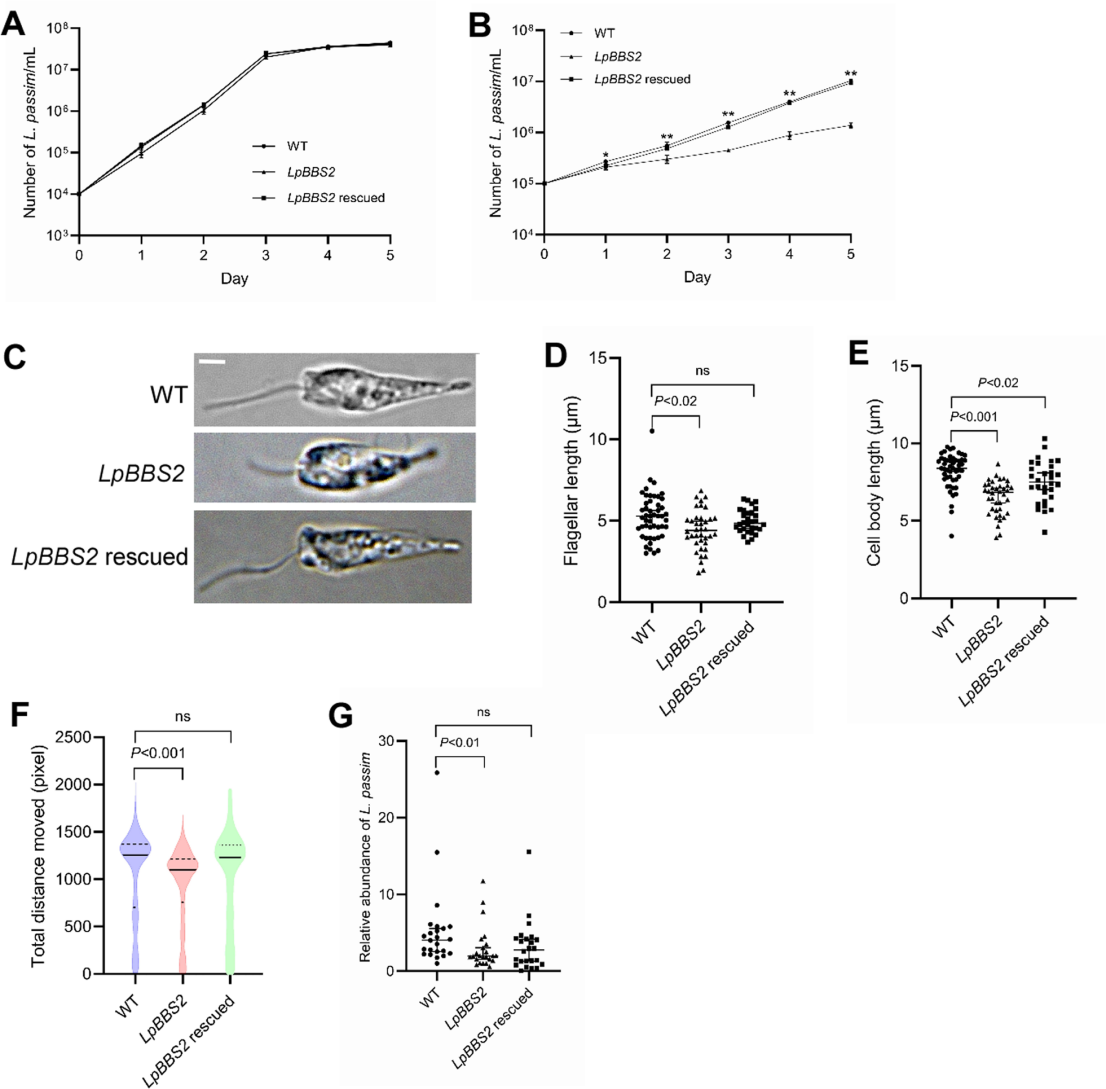
Phenotypes of *LpBBS2*-deficient mutants in culture and honey bees. **A** Growth rates of WT (circles), *LpBBS2* homozygous mutant (clone D8, triangles), and *LpBBS2*-rescued (squares) parasites were measured at 30 °C over 5 days (biological replicates, *n* = 3). **B** Growth rates of WT, *LpBBS2* homozygous mutant, and *LpBBS2*-rescued parasites were measured at 21 °C over 5 days (biological replicates, *n* = 3). Statistically significant differences in growth rates between WT and *LpBBS2* mutants across days 1–5 are indicated (asterisks, day 1; *P* < 0.02, day 2; *P* < 0.005, day 3; *P* < 0.001, day 4; *P* < 0.001, and day 5; *P* < 0.001, two-tailed Dunnett test). **C** Morphology of WT, *LpBBS2* homozygous mutant, and *LpBBS2*-rescued parasites. Scale bar: 2 μm. **D** Flagellar length comparisons between WT (*n* = 49), *LpBBS2* homozygous mutant (*n* = 38), and *LpBBS2*-rescued (*n* = 30) parasites. The median value, along with the 95% confidence intervals (CI), is shown. Flagellar length significantly differs between WT and *LpBBS2* mutants (*P* < 0.02) but not *LpBBS2*-rescued parasites (ns). **E** Cell body length comparisons between WT (*n* = 49), *LpBBS2* homozygous mutant (*n* = 38), and *LpBBS2*-rescued (*n* = 30) parasites. Cell body length significantly differs between WT and *LpBBS2* mutants (*P* < 0.001) as well as *LpBBS2*-rescued parasites (*P* < 0.02). **F** Motility (total distance moved in 1 min) of WT (*n* = 1415), *LpBBS2* homozygous mutant (*n* = 1178), and *LpBBS2*-rescued (*n* = 2385) parasites is shown via violin plot. Median, first, and third quartiles are indicated by solid and dashed lines, respectively. Motility significantly differs between WT and *LpBBS2* mutants (*P* < 0.001) but not *LpBBS2*-rescued parasites (ns). **G** Relative abundance of *L. passim* in individual honey bees (*n* = 24) 14 days post-infection, comparing WT, *LpBBS2* homozygous mutant, and *LpBBS2*-rescued parasites. Data is normalized to one sample infected with WT *L. passim* as 1, and the median with 95% CI is presented. Infection to honey bees significantly differs between WT and *LpBBS2* mutants (*P* < 0.01) but not *LpBBS2*-rescued parasites (ns). Statistical analysis was conducted using a two-tailed Steel test

Given the challenges in depleting LpBBS1, we focused on LpBBS2. Using CRISPR, we successfully disrupted the *LpBBS2* gene, replacing its partial open reading frame (1–1271) with *Hph* gene (Fig. 3A). Diagnostic PCR confirmed the loss of wild-type alleles at both the 5′ and 3′ ends of *LpBBS2* in the homozygous mutants (Fig. 3B). We used a reverse transcription (RT)-PCR to test for the presence of *LpBBS2* and *LpGAPDH* mRNAs. In homozygous mutants, *LpBBS2* mRNA was absent (Fig. 3C). In contrast, the amount of *LpBBS2* mRNA was similar in wild-type and heterozygous mutant parasites (Fig. 3C), indicating that *LpBBS2* is likely haplosufficient. Unlike LpBBS1, LpBBS2 was not essential for *L. passim* viability under culture conditions. We also established *LpBBS2-*deficient parasites, wherein *LpBBS2* gene was introduced on a plasmid DNA vector containing a bleomycin resistance gene (*LpBBS2*-rescued). Comparative phenotypic analyses were performed with wild-type and the parent mutant parasites. Growth analysis revealed that while the *LpBBS2* mutant grew normally at 30 °C (Fig. 4A), it exhibited dramatically reduced growth at 21 °C (Fig. 4B). These results suggest that cellular functions of *L. passim* BBSome without the BBS2 subunit become less effective at low temperatures. In the early stationary phase (3 days after culture initiation at 30 ℃), wild-type parasites displayed active movement with extended flagella. However, *LpBBS2*-deficient parasites had shorter flagella, smaller cell bodies, and reduced motility compared with wild-type parasites (Fig. 4C–F, Additional file 4: Supplementary Video 1, Additional file 5: Supplementary Video 2, and Additional file 6: Supplementary Video 3). The ectopic expression of *LpBBS2* successfully rescued the growth defects of *LpBBS2*-mutant parasites at 21 °C (Fig. 4B), as well as their flagellar length (Fig. 4C and D) and motility (Fig. 4F). However, it did not fully restore the smaller cell body size observed in the mutant parasites (Fig. 4E). Nevertheless, the cell bodies of *LpBBS2*-rescued parasites were significantly larger than those of the parent mutant parasites (Scheffe test, *P* < 0.02). These findings indicate that LpBBS2 plays a critical role in the morphology and motility of *L. passim*.

### LpBBS2 and lipid raft integrity

**Fig. 5:**
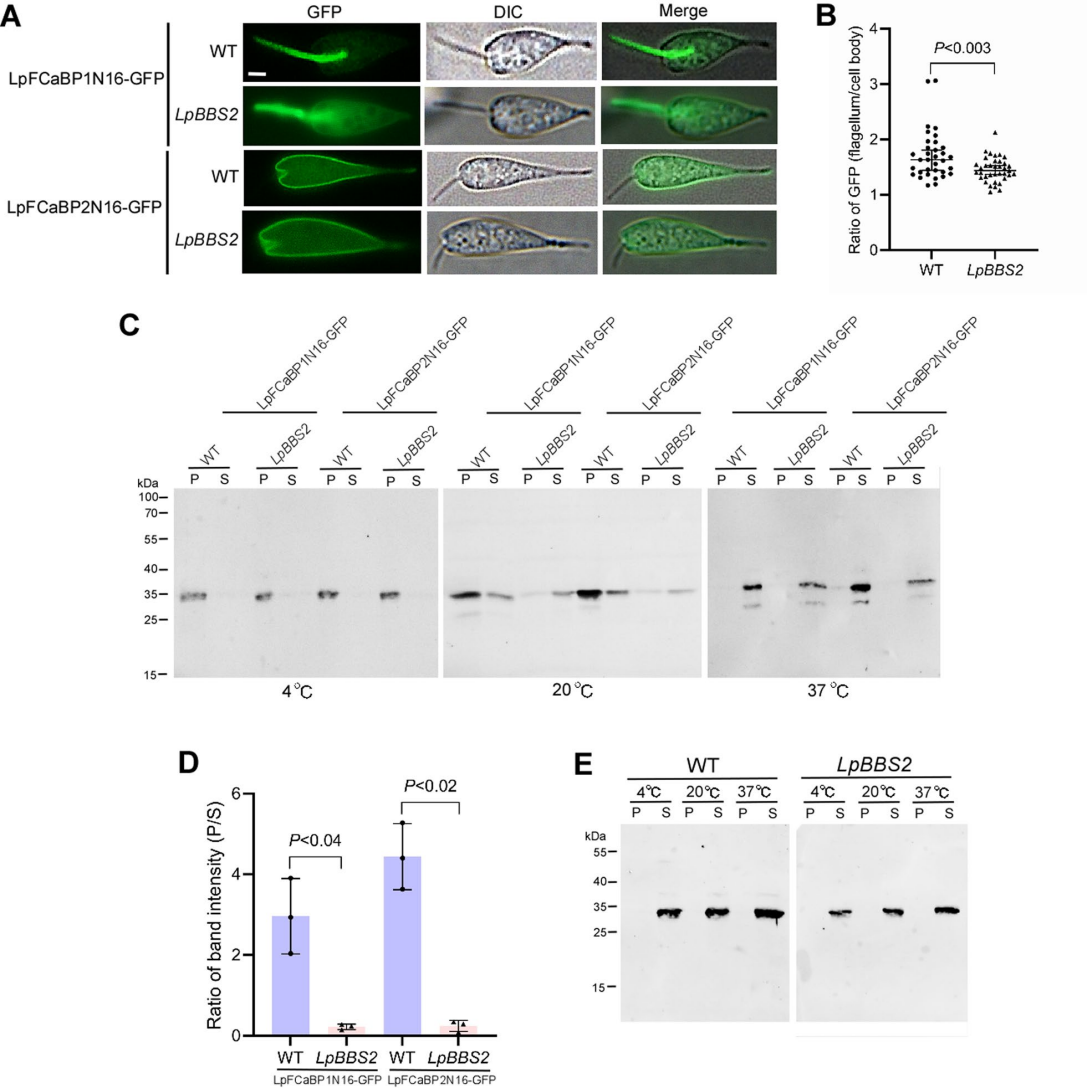
Localization and lipid raft association of LpFCaBP1N16::GFP and LpFCaBP2N16::GFP in WT and *LpBBS2*-deficient parasites. WT and *LpBBS2* mutant *L. passim* expressing LpFCaBP1N16::GFP or LpFCaBP2N16::GFP were observed under DIC and fluorescence microscopy. Merged images are shown. Scale bar: 2 μm. **B** Comparison of GFP fluorescence ratios (flagellum to cell body) between WT (*n* = 34) and *LpBBS2* mutants (*n* = 37) The median value, along with 95% CI, is shown, and statistical analysis was conducted using the Brunner–Munzel test. **C** WT and *LpBBS2*-mutant parasites expressing LpFCaBP1N16::GFP or LpFCaBP2N16::GFP were solubilized in 1% Triton X-100 at either 4, 20, or 37 °C, and the soluble (S) and pellet (P) fractions were analyzed by western blot using anti-GFP antibody. Molecular weights of protein markers are indicated on the left. **D** GFP band intensity ratio (P/S) from parasites solubilized at 20 °C is compared between WT and *LpBBS2* mutants (biological replicates, *n* = 3). The results are presented as the mean value ± standard deviation (SD). Statistical analysis was conducted using a two-tailed Welch’s *t*-test. **E** WT and *LpBBS2*-mutant parasites expressing GFP were solubilized in 1% Triton X-100 at different temperatures, and the soluble (S) and pellet (P) fractions were analyzed by western blot using anti-GFP antibody. Molecular weights of protein markers are indicated on the left

We previously reported that the flagellar calcium binding proteins (FCaBPs) localize in the flagellum (LpFCaBP1) and entire body (LpFCaBP2) membrane of *L. passim* through the specific N-terminal sorting sequences modified by myristoylation and palmitoylation. Indeed, the N-terminal 16 amino acids of LpFCaBP1 and LpFCaBP2 were sufficient to direct GFP to the flagellum and cell body membrane, respectively [43]. Given that the N-terminal 16 amino acid of LpFCaBP1 interacts with the BBSome, we examined the localization of LpFCaBP1N16::GFP and LpFCaBP2N16::GFP (GFP fused with N-terminal 16 amino acids of LpFCaBP1 and LpFCaBP2) in wild-type and *LpBBS2* mutants. In wild-type parasites, LpFCaBP1N16::GFP was enriched in the flagellum, but in the *LpBBS2* mutant, a significant amount was mislocalized to the cell body (Fig. 5A and B). LpFCaBP2N16::GFP remained in the cell body membrane of both wild-type and *LpBBS2* mutants. FCaBP was shown to associate with lipid rafts in the flagellum of *T. brucei* and *T. cruzi* [44, 45]. This is consistent with the idea that palmitoylation and myristoylation facilitate the association of the protein with lipid rafts [46]. We thus tested lipid raft association of LpFCaBP1N16::GFP and LpFCaBP2N16::GFP in wild-type and *LpBBS2*-deficient parasites. Because of their composition, lipid rafts are relatively resistant to solubilization with cold non-ionic detergents such as Triton X-100 and experimentally defined as detergent-resistant membranes (DRMs). DRM-associated proteins remain insoluble at 4 °C and thus pellet during centrifugation. However, they become soluble at higher temperature, 37 °C. Non-DRM associated proteins are soluble at any temperature [47, 48]. LpFCaBP1N16::GFP and LpFCaBP2N16::GFP were present in the pellet (insoluble) fraction when wild-type and *LpBBS2* mutant were extracted with 1% Triton X-100 at 4 ℃ but shifted to the soluble fraction at 37 ℃ (Fig. 5C). These results demonstrate that LpFCaBP1N16::GFP and LpFCaBP2N16::GFP associate with lipid rafts in both wild-type and *LpBBS2* mutant. When the parasites were extracted at 20 ℃, most of both proteins were present in the pellet fraction for wild-type, but they were in the soluble fraction for the *LpBBS2* mutant (Fig. 5C and D). Meanwhile, cytosolic GFP (non-DRM associated protein) was present in soluble fraction with both wild-type and *LpBBS2* mutant at any temperature (Fig. 5E). These results suggest that lipid raft association of LpFCaBP1N16::GFP and LpFCaBP2N16::GFP was weakened in the *LpBBS2* mutant at 20 °C. These findings suggest that LpBBS2 is involved in maintaining lipid raft integrity.

### Transcriptomic changes in *LpBBS2* mutants

**Fig. 6:**
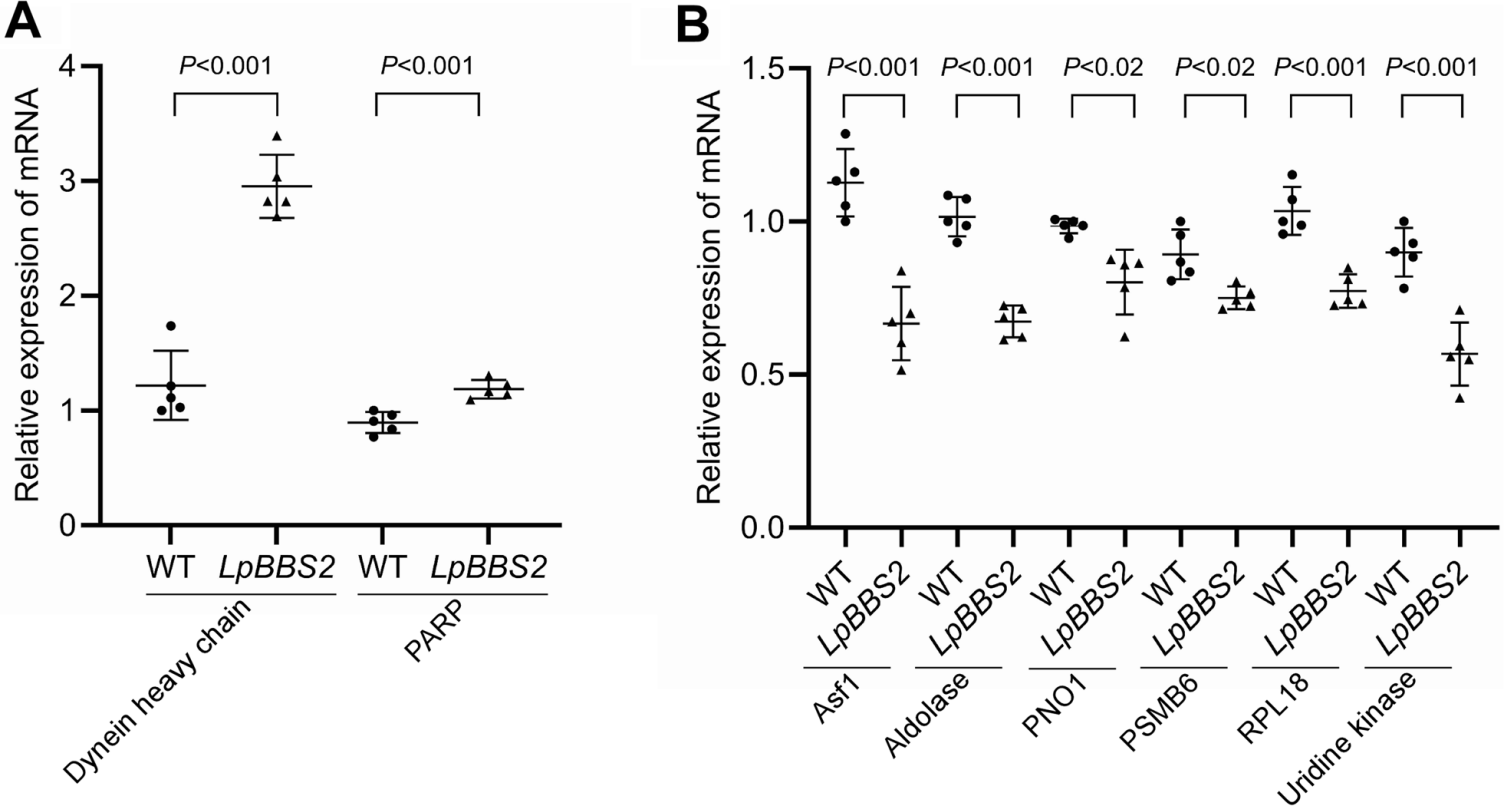
Quantification of differentially expressed mRNAs between WT and *LpBBS2*-deficient parasites by qRT-PCR. **A** and **B** Relative expression levels of dynein heavy chain, poly (ADP-ribose) polymerase (PARP), anti-silencing protein (Asf1), fructose-1,6-bisphosphate aldolase (Aldolase), pre-rRNA-processing protein (PNO1), 20S proteasome β6 subunit (PSMB6), 60S ribosomal protein L18 (RPL18), and uridine kinase mRNAs in WT (*n* = 5) and LpBBS2-deficient (*n* = 5) parasites. Data are normalized to one WT sample set as 1 for each mRNA and presented as mean ± SD. Statistical significance was determined using a two-tailed Welch’s *t*-test

To gain a more comprehensive understanding of the functions of the BBSome within the entire cell, we conducted RNA-seq analysis to explore the broader impacts of *LpBBS2* deficiency on the *L. passim* transcriptome. Principal component analysis revealed distinct transcriptomes for *LpBBS2*-mutant clones (D8 and G1) compared with wild-type (Additional file 7: Supplementary Fig. S1A). A scatter plot analysis showed differential expression of over 2800 genes between the mutants and wild-type. Expression of only 40 genes was significantly different between the clones D8 and G1, indicating their similarity (Additional file 7: Supplementary Fig. S1B–D). GO-term and KEGG pathway enrichment analyses suggested that upregulated genes in the *LpBBS2* mutant were associated with metabolism, cell cycle, cellular response to extracellular stimulus, DNA repair, cytoskeleton, mitochondria, and nucleus, while downregulated genes were linked to ribosome biogenesis and proteasome (Tables 1, 2, 3). Among genes related to IFT, we found that dynein light and heavy chain genes are upregulated in *LpBBS2* mutant (Additional file 8: Supplementary Table S1). Indeed, we found an increase in dynein heavy chain and poly (ADP-ribose) polymerase (PARP) mRNAs in *LpBBS2*-deficient parasites by qRT-PCR (Fig. 6A). PARP is a multifunctional enzyme that plays a central role in DNA repair, cell death regulation, transcription, and cellular stress responses. We also confirmed a decrease in mRNAs for anti-silencing protein (Asf1), fructose-1,6-bisphosphate aldolase (Aldolase), pre-rRNA-processing protein (PNO1), 20S proteasome β6 subunit (PSMB6), 60S ribosomal protein L18 (RPL18), and uridine kinase in *LpBBS2*-mutant parasites (Fig. 6B). Asf1 is a highly conserved histone chaperone involved in histone metabolism, chromatin assembly, and the regulation of transcription. These transcriptomic changes suggest that LpBBS2 plays a broad regulatory role in cellular processes.

**Table 1:** Representative GO terms enriched with genes upregulated in *LpBBS2* mutants

Ontology	ID	Description	Adjusted *P*-value
BP	GO:0051493	Regulation of cytoskeleton organization	0.024512417
BP	GO:1,901,360	Organic cyclic compound metabolic process	0.030397066
BP	GO:0033043	Regulation of organelle organization	0.031204177
BP	GO:0007346	Regulation of mitotic cell cycle	0.031204177
BP	GO:0006766	Vitamin metabolic process	0.031204177
BP	GO:0016311	Dephosphorylation	0.046513742
BP	GO:1,901,615	Organic hydroxy compound metabolic process	0.046513742
BP	GO:0031668	Cellular response to extracellular stimulus	0.049831369
BP	GO:0032886	Regulation of microtubule-based process	0.049831369
BP	GO:0051103	DNA ligation involved in DNA repair	0.049831369
BP	GO:1,901,700	Response to oxygen-containing compound	0.049831369
CC	GO:0005739	Mitochondrion	0.00011969
CC	GO:0005634	Nucleus	0.024513222
CC	GO:0005814	Centriole	0.045586455
CC	GO:0005874	Microtubule	0.045586455
CC	GO:0090734	Site of DNA damage	0.045586455

BP: biological process; CC: cellular compartment

**Table 2:** Representative GO terms enriched with genes downregulated in *LpBBS2* mutants

Ontology	ID	Description	Adjusted *P*-value
BP	GO:0006364	rRNA processing	0.000412333
BP	GO:0034470	ncRNA processing	0.000412333
BP	GO:0042254	Ribosome biogenesis	0.000412333
BP	GO:0022613	Ribonucleoprotein complex biogenesis	0.002349856

**Table 3:** KEGG pathways enriched in *LpBBS2* mutants

Upregulated genes		
ID	Description	Adjusted *P*-value
ko03029	Mitochondrial biogenesis [BR:ko03029]	0.000637691
ko05208	Chemical carcinogenesis—reactive oxygen species	0.023018084
ko04714	Thermogenesis	0.046239438
Downregulated genes		
ko03011	Ribosome [BR:ko03011]	2.05511E-23
ko03009	Ribosome biogenesis [BR:ko03009]	7.12255E-15
ko03051	Proteasome [BR:ko03051]	0.001109833

### LpBBS2 is essential for efficient infection in the honey bee hindgut

To assess the role of LpBBS2 in *L. passim* infection, we infected honey bees with wild-type, *LpBBS2*-deficient and *LpBBS2*-rescued parasites, and then measured parasite load in the hindgut after 14 days. The *LpBBS2* mutant exhibited a significantly reduced infection rate compared with wild-type (Fig. 4G), indicating that LpBBS2 is required for efficient infection of the honey bee hindgut. Infection of honey bees with wild-type and *LpBBS2*-rescued parasites was comparable.

## Discussion

### Application of AID in trypanosomatid parasites

AID has been successfully used to study gene functions in various organisms, from yeast to mammals [25, 26]. In this study, we applied AID to investigate the function of LpBBS1, which is essential for the viability of *L. passim*. The degron-tagged GFP at the N-terminus was rapidly degraded upon the addition of IAA; however, this was not the case for LpBBS1, which required prolonged incubation with IAA (more than 5 days) to sufficiently deplete the protein (Fig. 2). The degradation rate via AID appears to depend on both the target protein and the position of the degron sequence in *L. passim*, similar to findings in other species [25, 26]. Through AID, we discovered that LpBBS1 is necessary for the normal growth of *L. passim* under culture conditions. This differs from *L. major* and *T. brucei*, where BBS1 is not required for normal growth [21, 22]. Thus, the BBSome in monoxenous trypanosomatid parasites may have additional roles not present in dixenous trypanosomatid parasites. AID is a useful approach for studying gene function in trypanosomatid parasites under culture conditions; however, its application in vivo could be challenging, as IAA needs to be administered to the host and remain stable.

### Cellular functions of the BBSome in flagellated protists

In contrast to LpBBS1, we found that *L. passim* lacking LpBBS2 is viable. BBS2 and BBS7 subunits form the head of the BBSome by creating an asymmetric heterodimer, that connects with α-helical domains [3–6]. However, some species, such as fruit flies, lack the *BBS2* gene [49], suggesting that the head part is not essential for the core function of the BBSome. Alternatively, the BBSome subunit may have individual function without forming the octamer. Nevertheless, we found that LpBBS2 is necessary for the normal growth of *L. passim* in culture at 21 °C, but not at 30 °C. Furthermore, *LpBBS2*-deficient parasites have shorter flagella and cell bodies compared with the wild-type, and their motility is also reduced (Fig. 4). We observed that the ectopic expression of LpBBS2 does not fully restore the small cell body phenotype in *LpBBS2*-deficient parasites across two clones, D8 and G1. Since the expression of Cas9 and gRNA does not alter genomic DNA in *L. passim* [31], it is unlikely that the two *LpBBS2*-mutant clones have mutations in other genes. Consistent with the role of the BBSome in the IFT of membrane proteins, the dually-acylated LpFCaBP1N16::GFP was less abundant in the flagella of *LpBBS2* mutants compared with the wild-type. Although the BBSome does not seem to be involved in the IFT of membrane proteins in *T. brucei* and *L. major* [21, 22], it plays an important role in other flagellated protists. In *Chlamydomonas reinhardtii*, the BBSome is required for the ciliary export of dually-acylated phospholipase D via retrograde transport [14]. In *Paramecium tetraurelia*, RNAi knockdown of the BBSome results in the loss of calcium-activated K^+^ channels and PKD from the cilia [50]. In *Euglena gracilis*, the deletion of BBS7 and BBS8 leads to the loss of flagella [51]. The weak association of LpFCaBP1N16::GFP and LpFCaBP2N16::GFP with lipid rafts suggests that the lipid raft environment may be altered in *LpBBS2* mutants. Since lipid rafts in trypanosomatids are rich in 3 β–hydroxysterols and sphingolipids, similar to those in other species [45, 52, 53], enzymes involved in the metabolism of these lipids may be affected. Alternatively, the lipid rafts associated proteins necessary to anchor FCaBPs would be changed in the mutants. Although LpFCaBP2N16::GFP is highly enriched in the cell body membrane of *LpBBS2* mutants (Fig. 5A), we can not completely rule out the possibility that the DHHC palmitoyltransferases and N-myristoyltransferase, responsible for palmitoylation and myristoylation, are partially impaired. Protein acylation is known to be critical for the association with lipid rafts [46].

We believe the transcriptomic changes in *LpBBS2* mutants are likely direct or indirect consequences of compensating for the lack of this gene. Since dynein is the specific motor protein for retrograde transport along microtubules [54], the upregulation of dynein light and heavy chain mRNAs in *LpBBS2* mutants would be consistent with the role of the BBSome in IFT of membrane proteins. According to GO terms enriched with upregulated genes in *LpBBS2* mutants, the BBSome may play multiple roles related to metabolism, DNA repair, plasma membrane protein localization, mitochondria, and microtubules in *L. passim*. Indeed, many studies report these non-ciliary functions of the BBSome. For example, defective cell division, migration, and adhesion in BBS4-deficient cells indicate a role for the BBSome in controlling cytoskeleton [17]. The BBSome is also required for the plasma membrane localization of proteins such as the leptin receptor [16], Notch [55], and the insulin receptor [56], suggesting a role in intracellular vesicular trafficking. The BBSome was shown to interact with proteasomal subunits, and the loss of BBS4 depletes several subunits from the centrosomal proteasome, leading to the accumulation of specific proteins [19, 57]. This role in protein degradation is relevant to our findings that genes involved in the proteasome and ribosome pathways are downregulated in *LpBBS2* mutants (Tables 2 and 3). The BBSome has also been shown to regulate mitochondria by affecting the phosphorylation and mitochondrial translocation of dynamin-like protein 1 [20].

We found that *LpBBS2*-deficient parasites are ineffective at infecting the honey bee hindgut. Since LpFCaBPs-deficient parasites, which have short flagella and low motility, can still infect the honey bee hindgut [43], it is unlikely that the low infectivity of the *LpBBS2* mutants is due to their morphological or motility defects. We speculate that the alteration of lipid rafts in *LpBBS2* mutants may be responsible for the ineffective infection. Lipid rafts in trypanosomatids appear to play roles in signal transduction, virulence factor function, and endocytosis/exocytosis [58]. In the mutant, the localization and regulation of signaling molecules that may be critical for parasite adaptation to the honey bee hindgut would likely be altered [59]. For instance, the GPI-anchored variant surface glycoprotein, essential for immune evasion, is associated with lipid rafts in *T. brucei* [60]. This is also true for the virulence factor GP63 in *L. major* [60]. Furthermore, lipid rafts are implicated in endocytosis and exocytosis, which are crucial for nutrient uptake and surface protein recycling [61]. These are vital processes for parasite survival and infection. However, the role of LpBBS2 in lipid rafts may not be shared with *Leishmania* spp., as *BBS2*-deficient *L. mexicana* can reach the anterior gut and survive in sand flies [62]. The *L. passim* BBSome appears to have diverse cellular functions and some of them are not shared with the related trypanosomatid parasites. BBSome appears to have the ability to be an adaptor to connect any of multiple proteins in a species-specific manner under various cellular contexts.

## Conclusions

BBSome was shown to play a major role in the ciliary transport of membrane protein, but it also has many non-ciliary functions, including intracellular vesicular transport, cytoskeletal dynamics, gene expression, and cellular and organelle homeostasis. To gain insight into the ancestral function of the BBSome and its potential co-option into diverse cellular functions, we focused on the BBSome in a trypanosomatid parasite, one of the earliest-diverging flagellated protists among eukaryotes. We aimed to answer the above question by exploring the functions of the BBSome in *L. passim*, a monoxenous trypanosomatid parasite that infects honey bees, and comparing it with previously studied dixenous trypanosomatid parasites, *Leishmania* and *Trypanosoma*. Our results suggest that while the BBSome has multiple cellular functions in a monoxenous trypanosomatid parasite, some of these functions are not shared with related dixenous trypanosomatid parasites. We propose that the BBSome has the ability to act as an adaptor, linking multiple proteins in a species-specific manner under various cellular contexts.

## Supplementary Information

Additional file 1: Supplementary Dataset 1. Component of modified FP-FB medium

Additional file 2: Supplementary Dataset 2. DNA sequences of *LpBBS1*, *LpBBS2*, and *LpIFT88*

Additional file 3: Supplementary Dataset 3. List of primers used in this study

Additional file 4: Supplementary Video 1. Movement of wild-type *L. passim* was measured at 3 days after culture at 30 °C (Fig. 4A). To track the movement of parasites, we created video by capturing images every second for 1 minute. The total distance traveled was plotted in Fig. 4F.

Additional file 5: Supplementary Video 2. Movement of LpBBS2-deficient *L. passim* was measured at 3 days after culture at 30 °C (Fig. 4A). To track the movement of parasites, we created video by capturing images every second for 1 minute. The total distance traveled was plotted in Fig. 4F.

Additional file 6: Supplementary Video 3. Movement of LpBBS2-deficient *L. passim* with ectopic LpBBS2 (LpBBS2 rescued) was measured at 3 days after culture at 30 °C (Fig. 4A). To track the movement of parasites, we created video by capturing images every second for 1 minute. The total distance traveled was plotted in Fig. 4F.

Additional file 7: Supplementary Fig. S1. Comparison of RNA-seq data for WT and LpBBS2-deficient parasites. (A) Principal component analysis of RNA-seq samples from WT (blue), LpBBS2 mutant clone D8 (green), and clone G1 (beige). (B-D) Scatter plot analysis showing upregulated (red), downregulated (blue), and non-significantly different genes between WT and clone G1 (B), WT and clone D8 (C), or clone D8 and clone G1 (D).

Additional file 8: Supplementary Table S1. Normalized read counts of dynein heavy and light chain mRNAs in wild-type and *LpBBS2* mutants.

## Data Availability

All data are included as tables, figures and Additional files in the article. The RNA-seq data were deposited to NCBI with the accession numbers listed in the article.
