# Supplementary material for: BBSome deficiency in Lotmaria passim reveals divergent functions in trypanosomatid parasites
Source: Parasit Vectors. 2025 Feb 18;18:60. doi: 10.1186/s13071-025-06704-3 (PMC11837635; doi:10.1186/s13071-025-06704-3)
Supplement: Supplementary file 1 — Additional file 1: Supplementary Dataset 1. Component of modified FP-FB medium [file 13071_2025_6704_MOESM1_ESM.docx]

**Components of culture medium for *L. passim***

| **FP stock solution**  NaCl 2.8 g  KCl 0.4 g  NaH_2_PO_4_ 8.875 g  Tryptose Soya Broth (BIODEE) 10 g  Brain Heart Infusion (HANGWEI) 2 g  Adjust the pH to 5.8 and add water to a final volume of 1000 mL  **10×FB stock solution**  D(-)Fructose (BIODEE) 1.8 g  L-Proline (BIODEE) 0.289 g  Vitamine B1 hydrochloride (BIODEE) 6.7 mg  Folic acid (Biosharp) 4.4 mg  Add FP stock solution to a final volume of 100 mL  **Haemin stock solution**  Haemin-chloride (BIODEE) 100 mg  1M NaOH 1.25 mL  Dissolve by heating and add water to a final volume of 50 mL |
| --- |
| **Complete medium**  FP stock solution 36 mL  10×FB stock solution 4 mL  Haemin stock solution (BIODEE) 44.5 μL  Heat-inactivated fetal bovine seru 4.5 mL  100×Penicillin-Streptomycin solution (Beyotime) 450 μL |
