# Supplementary material for: BBSome deficiency in Lotmaria passim reveals divergent functions in trypanosomatid parasites
Source: Parasit Vectors. 2025 Feb 18;18:60. doi: 10.1186/s13071-025-06704-3 (PMC11837635; doi:10.1186/s13071-025-06704-3)
Supplement: Supplementary file 2 — Additional file 2: Supplementary Dataset 2. DNA sequences of LpBBS1, LpBBS2, and LpIFT88 [file 13071_2025_6704_MOESM2_ESM.docx]

**Dataset S1**

>LpBBS1

ATGGCGCAGAAGGAAAAAAGCAAAGGGGAGTCGAAGGAGAAGTTCTGGTTGTACGCCTTCCGCGACCACCTCGCCAACCTGCGCGCTTTTTCGAACTGTATCGAGACGGCCGACGTCAGCGGCAACGGCGACTACCAGCTGCTCGTGGCAGATGGCAGCAAAAAACTAAAGGTCTTCGGCGGCACCGCCCTGCAACGCGAGTTGCCTCTCTTTGGCGTGCCGTCGGCGATCGCCTCCTTTTACATGAGCACCAATGACGCCTTCAACAAGCCAGTAATCGCAGTGGCGACGGGGCCGTACATCTTCATGTACCGCAACAACAAACCTCTCTATCGCTACATGATTCCCGCCGTCCCGATTGACGCGCAGGAGTCGGATATTTGGAAAAAGCTCGCTGACGGCGTCTACACCGTCGAGGACGCCGTGGCGAAGTTGGAGTCGCTGCTCGACTCAGGTGTGCAGACCTCGTCGCGGACGCTGGAGTTGCTGCTGCTAGACACGGAGGAGGAGCGGACTGACTTTGTGACGCGTATGAGCGCCATCCCGCTCATTCAGATGGATGTGGCGACTTGCATGACGTCAATCCCGCTGGAGACACTGGAGGCAGAAGGCACGAGCTGCCTGGTCGTGGGTACCGAGGCGTGCTTTCTCTATGTCTTGGGGGCCGCGACGATGGAGGTGTCGCTGAAGGTGGTGCTGCCGAGCCCGCCGGTGTTTTTAATCGTGGCCGGCTGCTTTGCCGTGGATTACCGCATCATCATCGCATGTCGCGACGGCCGCGTTTACTCCATCAAGCACGGCCACCTGCACAGCGCTGTCATCCAGCCTGACGCGCAGCCTTGCGCCGTGGCTCGCTTCGGCAACTTGATCGCCGTTGCCACCACCGCCAACACGCTCACCTACTACAATCTGAAGGGCAAGAAGCAGCAAAGTCTGTTCCTGCCGTGCCCCATCACGAACTTGACCACCATCACTGACCCGATCACTGGAGAGGACAGGGGCCTCGTCGTCGCCCTCAGCAATGGCGAGATTCGCGTGCTGGTCGGCACGCAGCTGCTGCACGTGAGCCTCGTGTATGGTACGGTGACAGCCATGAAGTTCTGCCGCTACGGCCGCGCGGACGGGGCCCTCATTCTCGTCCTGCAGAACGGCTCGCTCGTCGTCGAGCTGCTGCACCGCAACGCCGACCTCACCTCCAGCAAAAAGGTGGAGACGGGCCCGCCACCGGAGCAGGACGTGCCGATCCCCGTGCCCTTTCTCAGCTCTGTCTTTACGGCGCAGACGTCGCGGGAGCGCAAGTACGGCGCGGACATGTACCAGCTTTTCCAGTACGACCTCTCGCAGCTGCGCTTGACAGCGGCAAAGGCGTATTTGGAGATGGTCGGTAGCGGCGCGGTGCCGACGGAGCTGGGCAACGTGACGGAGGAGAACGAGGAGGTGGCGGAGTCGTCCCTGCGCATGAATACTGTGGTGCAGGGGCTTGGGCCCGTCTTTAAGGTGAAGGTACAGCTGCAGAACATCGGTGCGGCGCCGCTGCATGCCGTCCGGGTGGTCTTCTGCCTCTCCGACGACGATATGTATCGCATGCCGCAGCAAGTTTTCACGATTCCGACGTTGCTGCCCTCCGTTCCGCTGTCATGTGAGGCGCTGGTGGAGTTGGTGGAAGGGGAGGTCAAGGGCAACGCAATTTTGGTCGTCGCCTCAGAGCCGAAGAGCACCAATCCGCTTGCAAGCACGTTGGTAGACCTTCCCGAGGCGGAGCTGATTGAGGGGCTGTAA

>LpBBS2

ATGGCGTCTACCGCGGATTCAGACAACCACATACGCCTTAGCACTGCGTTTGAGCTCAACATTGGCGCCCCGATTCTCGTCGACCGCGTCGCAGCAGGCCGCTTTCTGCGCAATGCCACCGCGCGTGAGGCAGCGTTGGCGGCGCACGCCACCCGCTGTCCCCCGCCTGCCGTCTCGCTCGCCTTCGGCAGCAGCGGCCAACGGGTGGTGATTCACAACAACAACAACAGCACCGCCGAGGCGAAGCACTCGGCGAAGCGGGGGGAGGCGCAGGCGAGCGCGGGAGCTGCCATCGGCGCCACCGAAGACACCTCCCTGCAGACCCTCAGCTTTGGCAAGGCGCCAACGGCACTGGCGGCTGGTCAGCTCTATAGTCCGGACAGCACCGAGGTGGAAGCCAGCTACGATGCGCTGCTTTTTGGTGCAACGACGAGCCTGCTGGCCTACGATGTGGAGCAGAACAAGCAGCTCTTCTACAAGGACGTGGAGGATGGAGTACAGGCGGTCGTGTGCGGGCCCGTCATGAAGCCGCAGGATTCGAGCGCGGATGCACCCCCGCTCGCCATCGTCGGAGGCAATTGCAGCATCTTCGGCTTCGACCGCACGGGAAGCGAACGGTACTGGACCGTGACGGGTGACCAGGTCACGGCAATGGCGCTGATGCCGTGGTCGGCGCCAACCGTCGCCGCCACACTGTCTCCCTCTTCGTTCTCGCCGCTCACCCTCTTAGCGGCTTCGGAAGATTTCGAGATACGCGCCTACGCGGGCGAGGAGGCGATGGCGACAATACACGAGGTGGATAAGGTACGGAAGCTAATTCCTCTGTGGCGACCACTGCGCACCGCAGCGGGAGGCGAAGAGGGGACCGGCGCTGACGGGCGTTTTGGCCGCTTCGCCTATCTGCTGGAGAACGGCACCGTCGGTGTGTACGAACGCGGGGAGCGGGTGTGGCGTGTAAAAGGCAAGTCGACGCCCGTATCGGCCGCCTTCTGCGACGTGGACGGCGATGGCGTGGAGGAGTTGGTGGTCGGGTGGCAGAGCGGCCGTGTTGAGGTGCGTACGGACCGCGGCGGTAGCCTTGATAAAGGTGGCGCAGTGCTCTACCGCGACACCTACGCCACCGCGGTGGCTGCCGTGCTGGCGGAGGACTACCGGCAGGACGGAACAACACTGCCGCTGGTGTGCACCGTGGACGGCACCGTGCGGGGGCTGCGGCTGCTCGAGACGCAGCGCGAGGAGGCGGCGGAGGTGCGGCAGCTGCAGGTGCTGGAATCCCTTGCACAGGAGAAGGAGCAGCTGGCTGCTCAGCTGACCAGCCTGGAGGAACAGCTGGCCCGCCGTGCTGCGGGGGAGCAAGACACGACGATGCCAGAGTCGGGAGTAGAGGTGCACGGCCGTTGCGCTGCCAACTATGTCACGAAGCAGGTTGACGTGCACGTGGAGGTGTGCGGGTCGGCTGCCGAGCGTGGCGATCTTGTCGTGCACAGCTGCTTGTTGAAGTGCGATGCGTGGAGTGCGACGGACCATGACGTGATGGCCTTCACCGCAGCGGAACCTGGCACAACGCTCACCTGCAGCTTCGACCACCCAGACGACCTCCCGCTGCTGGTAACCGCCTTCGTGGCGGTGGGGCCGCCCTTCGCGGAGAACTACCAAATCCACGAGCTGGAGCTGCGGGTGCCGCGCTTCATCATGTACGCGCTTCCCCACACGTTGCAGCAGACGTCGGAGGCAGCGGCACAGATGGGCGGTTCACTGGCCTATGTCCCGCCCACCGGCTACGTGTCGCTGCGCTGGCGCGACGCGCTGAAGCTTGACGTGATCGAGCAGTGGCTGCGGCAGTCGTTCAGCGTTCCGGAGGAGTTGAGTCTCTCCGACGCGAACTCGCCCGAAGACCCGGTGCTCCATCTCGAGCTGCTTCACGTGCGCGACGGCAGCAGCCTCGGAATAGAGGTGCGCAACAGCACCGCGGCCACCGGCGACGCCTTCAGTGTCTTCACACTGCGCTCGGACCATTTGGCTCCCTGCGGGGAGGTGGTGAATGCCTTCGCGGAGGATTTGAAAGGTGCCTGGGCGGACCCGCCGTCAGACCCCGTCGAAGTGCGATGTGAGATGGGCGCAGAGTTGGAGCGGCTACGTGGCGTCTTGGCTCGCGTGGACGAATTTAACGAGGTGCGCATGAAGCTGACGACCGACATGGCGGACGCGGCAACCATCGTCAAGACATTGCTCGGCCGTGCCGAGGACGCGCGACTGCTCGGCGACATGACCTCCATGAAAAAGTCCTACGCGGCCCTGTACGACGTGGACCAGGAATTGCTCGGCGAGAACGCGAAACGGATTAGCAACTACGAGGAGTTGAAGTTGGCGCTGAAGGAGGTAAACACGGCGATCCAGCACGCTGGGAAGCTGCGCATCGGCCCAGCGAGAGCGCAGCTGGTCGCGGACTGCCGCAACGCCTTGAAGGAAAACAAAGTGAACAGCTTGCTAGAGATCATCCGCACCGGGTCTGAGTAG

>LpIFT88

ATGACGACCAGCAACGACGATATCTACGCCGCCTTCCAAACACCGGAGAGCGGCGCCAACCCCTGGACGACGAGCGCCAATCCCTTTGAGGCCCCGCCGACCGAGTCGCTTGGCGGCAACCCACTCATGCAGGCCCCGCCGTCCCAGTGGGGACGCACCGGCATGGGCTCCGCGTGGGGCGTGCCGGGCAGCCGCCTGGGCACGATGGGCGGTGCTCTCGCTGGTGCGGCGCGGCCCATGACGTCAAACCGCCCGGTCGGCTTCAGCAGCTCACCGAAGGCCGGCGCGGCAGCGCTCTTCGACCCGACCGGACAGGCCCGCAACGCGAGCATGGCAGTAGGCCCGGCCCCCCCGCTGAAGAAGCGTAGCGAGAACAGCCCCGAAGAGGAGTTCGCCGAGATGGAGAGGCAGGTGAACAAGCTGATCGAGGAGAGCGCCATGCTGGCCAAGCAGAAGGACTACGGCGCGGCGCTGGAGAAGGCGAAGGACGCCGGCAAACTCGAACGGCAGCTGTGCAAGCAGCGCGAGCAACGCGGGTTTGCAGAGCAGATCAACGTCGACCTCACCTACGCCGTGCACTTCAACCTAGCAGTGCAGTACCAGAACCACCAGCTGTACAACGAGGCCCTCAACACGTACAACCTAATCATCCGCAACGTGCAGTTCCCGCAGGCGGGGCGGCTGCGTGTGAACATGGGCAACATCTACCTCGCCCAGAACAACTACCTGCTTGCCGTCAAGATGTACCGCGCCGCGCTGGACGAGGCGCCGGCGGCCGGCAAGGAGCTGCGCTACCGCCTGTGCCGCAACATCGCGAACGCTTTTGTGAAGCTGGGCCAGTACCGCGACGCGGCGAACAGCTACGAGACGGTGGTGGAGGGAAACTCCGACATGACGTCCACCTTTAATCTCATCCTTTGCTATTACGCGTTGGGGGAGACGGAGAAGATGAAGCGCACCTTCACGCGGCTCCTCAGCTGCCAACTTGCCGGCCTCGACGGCGAGGAGGACTTCGAGGAGGAGGAGAAGCGGAAGGACGTGCTGGTCGACGACGGCCTCAGCCGCATGCGCAAAGAGCGGCGGGCCCGCTACCTGCAGTACATCATCACCGCCGCCCGCCTCATCGCCCCGGCGCTGAACAAGGACTGGTGCGTCGGCTATGACTACATCATTGGCCAGCTGCGCAACTACGAGATGCGCGACCCGAGCTCGCACGTGGCGAGCGAGCTGGAGATGTGCAAGAACCTGAACTTTCTCAAGCACAAGCGCTACCAAGAGGCGATCAACGGGCTGAAGCTGTTCGAGAAGAAGGACAAGAGCCTCCGCGCGCGCGCGGCGACGAACCTGGCGTACCTCTACTTTCTCGAGGGCGACTATGATAGCGGGGAGCAGTACAGCGACATGAGCCTGGAGGAGAACCAGTACAACGCGAAGGCGCTGGTGAACAAGGGCAACTTTGCCTTTGTGAAGAAGGACTTCGAGAAGGCGAAGGAGTTGTACAACAAAGCCCTCGCGGTCGAGGCCGACAACGTCGAGGCAATTTACAACCTCGGCCTCGCCGCAAAGAAGCTGGGCCTCTACGAGGAGTCGGTGCGCGTGTTCAAGCGGGTGCAGGCGCTCGTGGATAGCAGTGAGGTGCTCTACCAAATTGCCGACCTGAACGACCTCGTCGGCGACCCGTCCGCGCTGGAGTGGTTCAACCGGCTCATCGGACGCGTGCCGACAGACCCGAACGCGCTGGCCCGCGTCGGCTCCCTCTACGCCCGCGACGGCGACGACGTGCAGGCATTTCACTACTACCTCGAGGCGTACCGGTATTACCAGGTGAACATGGACGTCATCTCGTGGCTGGGCGCCTACTTTGTGAAGAACGAGGTGTACGACAAGGCGGTGCAGTTCTTCGAGCGCGCGTCACACATCCAGCCGCAGGAGGTGAAATGGGAGCTGATGGTCGCTTCTTGCCACCGCCGCCGTGGCGACTACGTACTGGCAAAGCGCCTCTACGAGCAGGTGCACCGCAAGTACCCAAACAACATCGAGTGCCTCAAGTACTTGGTGCAGATCTGCAAGGACGCGAACCTTGCGGAGGAGGCGAATGAGTGGTTCAAGGCGATGAAGAAGGTGGAGCGACAGCAGATCCATAGCAGCAGCAGCAGCGTCGCTGGCGAGAGCGGCGACGACGACGACTCATCGGACGGTGGCAACAACCGTCGCGGCAGCACGGCGACGAATGGCGGCACGAGCAGCGAGGCCCCGGTGGCGGGTCGCCGCGCTGGTGGGGGTAACGCGGCGGTCGACAAGGACCTCACGGTCGGATTGTCCGACGACGACATGGTGGACAACAAGAAGAAGAGCAACGTCAATGGCAAGGCGAAGAAGAAGGAGAGCGACTCGGATGAGGATATCGACCTTCCCGGCATTTAA
