# Supplementary material for: BBSome deficiency in Lotmaria passim reveals divergent functions in trypanosomatid parasites
Source: Parasit Vectors. 2025 Feb 18;18:60. doi: 10.1186/s13071-025-06704-3 (PMC11837635; doi:10.1186/s13071-025-06704-3)
Supplement: Supplementary file 3 — Additional file 3: Supplementary Dataset 3. List of primers used in this study [file 13071_2025_6704_MOESM3_ESM.docx]

**Dataset S2**

| Primer | Sequence (5’→3’) |
| --- | --- |
| 3Myc-N-5 | CTAGAATGGAGCAGAAGCTGATTTCTGAGGAAGATCTGGGCACAGGATCCGAACAGAAACTGATTTCTGAGGAAGATCTGGGCAGCGCCGGAGAGCAGAAGCTGATTTCTGAAGAGGATCTGA |
| 3Myc-N-3 | AGCTTCAGATCCTCTTCAGAAATCAGCTTCTGCTCTCCGGCGCTGCCCAGATCTTCCTCAGAAATCAGTTTCTGTTCGGATCCTGTGCCCAGATCTTCCTCAGAAATCAGCTTCTGCTCCATT |
| LpBBS1-5-HindIII | TTTAAGCTTATGGCGCAGAAGGAAAAAAGCA |
| LpBBS1-3-ClaI | TTTTATCGATTTACAGCCCCTCAATCAGCTCC |
| LpBBS2-5-ClaI | TTTTATCGATATGGCGTCTACCGCGGATTCAG |
| LpBBS2-5-XbaI | TTTCTAGAATGGCGTCTACCGCGGATTCAG |
| LpBBS2-3-ClaI | TTTTATCGATTTACTCAGACCCGGTGCGGATG |
| LpIFT88-5-XbaI | TTTCTAGAATGACGACCAGCAACGACGATATCTA |
| LpIFT88-3-XbaI | TTTCTAGAAATGCCGGGAAGGTCGATATCCTCAT |
| miniIAA-5-XbaI | TTTCTAGAATGGGCTTCTCTGAGACCGTGGACCT |
| miniIAA-3-XbaI | TTTCTAGAGGAGCTTGTCTTCTGCTGGGTCAT |
| AtAFB2-5-XbaI | TTTCTAGAATGAACTACTTTCCCGATGAGGTC |
| AtAFB2-3-HindIII | TTTAAGCTTTACAGAATCCAGACAAAGGGGGG |
| LpBBS1gRNA3’UTR48F | TTGTGAAAACAACAACAGTAAACG |
| LpBBS1gRNA3’UTR48R | AAACCGTTTACTGTTGTTGTTTTC |
| GAPDH-HindIII | TTTAAGCTTCGAGCCATTTACGACTCCAAGG |
| GAPDH-3-Hyg | CTTTTTCATATTGGCTGCAGGGTCGCTCGGTGT |
| Hyg-5-GAPDH | GCAGCCAATATGAAAAAGCCTGAACTC |
| Hyg-3-EcoRI | TTTTTGAATTCTATTTCTTTGCCCTCGGAC |
| LpBBS1-3’UTR-F-degron | TTTTTGAATTCGCCTGTAAATTACTCTACTTTGC |
| LpBBS1-3’UTR-R | TTTTTGAATTCCGAGTAAGTACTCTACCACCCGTA |
| LpBBS1-3’UTR-F-degron | CTAGAATGGGCTGTGCTTCTTCCCTGTTTTCCTCCATTTCCAAGACAGAGGATT |
| miniIAA7-5-HindIII | TTTAAGCTTGGCTTCTCTGAGACCGTGGAC |
| miniIAA7-3-XhoI | TTTTCTCGAGTTAGGAGCTTGTCTTCTGCTGGGT |
| LpBBS1-5-XbaI | TTTCTAGAATGGCGCAGAAGGAAAAAAGC |
| LpBBS1-3-XbaI | TTTCTAGACAGCCCCTCAATCAGCTCCGCCT |
| LpBBS1-5-degron | TTTTCTCGAGAGCCCGCCGGTGTTTTTAATC |
| miniIAA7-3-ClaI | TTTTATCGATTAGGAGCTTGTCTTCTGCTGGGT |
| LpBBS1-1681F | AACGCAATTTTGGTCGTCGCCTCA |
| LpBBS1-3’UTR-down | CATGGCGCAGTATGAAAAGCACTA |
| Hyg-846F | CGTATATGCTCCGCATTGGTCTTG |
| LpBBS2gRNA1091F | TTGTGCACTGCGCCACCTTTATCA |
| LpBBS2gRNA1091R | AAACTGATAAAGGTGGCGCAGTGC |
| LpBBS2 5’UTR-F | CTTGCTGTCACGGCGAATTGTAGA |
| LpBBS2 5’UTR-R | CTTTTTCATGTACCTCTACTGACCTTCTTTTTCTT |
| LpBBS2 Hph-F | TAGAGGTACATGAAAAAGCCTGAACTC |
| LpBBS2 Hph-R | CAAGGGATTCTATTTCTTTGCCCTCGG |
| LpBBS2 3’ORF-F | AAGAAATAGAATCCCTTGCACAGGAGAAGGAGC |
| LpBBS2 3’ORF-R | GACTCAACTCCTCCGGAACGCT |
| LpBBS2 5’UTR-Up | CTCATTCCAATCAAAGCACGGAGG |
| LpBBS2-47R | GCAGTGCTAAGGCGTATGTGGTTG |
| Hyg-159R | GCAGCTATTTACCCGCAGGACATA |
| LpBBS2-1216F | CTGCTCGAGACGCAGCGCGA |
| LpBBS2-1910 R | AGATGGAGCACCGGGTCTTCG |
| LpSL-F-1st | CTAACGCTATATAAGTATCAG |
| LpBBS2-70R | GGGCGCCAATGTTGAGCTCAAA |
| LpSL-F-2nd | ATCAGTTTCTGTACTTTATTG |
| LpSL-F | CTAACGCTATATAAGTATCAGTTTCTGTACTTTATTG |
| LpGAPDH-R | GGGCCAGGCAGTTGGTCGTG |
| LpITS2-F | GGGTCTTTTGTGATCGGGATAA |
| LpITS2-R | CAAAAAGATGCCTAACGTGAAGAA |
| AmHsTRPA-F | TAGCGTACATGTGGTGCTGT |
| AmHsTRPA-R | GCTAGGCTCCACGTAATCCA |
| L. passim 18S-F | GAAAGGAACCACTCCCGTGT |
| L. passim 18S-R | GTCCCGTCCATGTCGGATTT |
| LpDHC-R | TTCGCCTGCAGCTGTGATGTA |
| LpPARP-R | AACAGTAGCCTTTGGCGCAGC |
| LpAsf1-R | CTTCCAGTCGCATTCTCCACT |
| LpAldorase-R | CATCTTCTTCACCGTCGCGAT |
| LpPNO1-R | ACCTTCTTGAGCGGAGGAAAG |
| LpPSMB6-R | CCTGATATGGGCTCCATTGCT |
| LpRPL18-R | GCCAGGAACTTGTACAGCTTG |
| LpUridine kinase-R | CAGGTGCTCATCAGGTCTGTT |
