# Supplementary figures and images for: BBSome deficiency in Lotmaria passim reveals divergent functions in trypanosomatid parasites
Source: Parasit Vectors. 2025 Feb 18;18:60. doi: 10.1186/s13071-025-06704-3 (PMC11837635; doi:10.1186/s13071-025-06704-3)

Figure S1

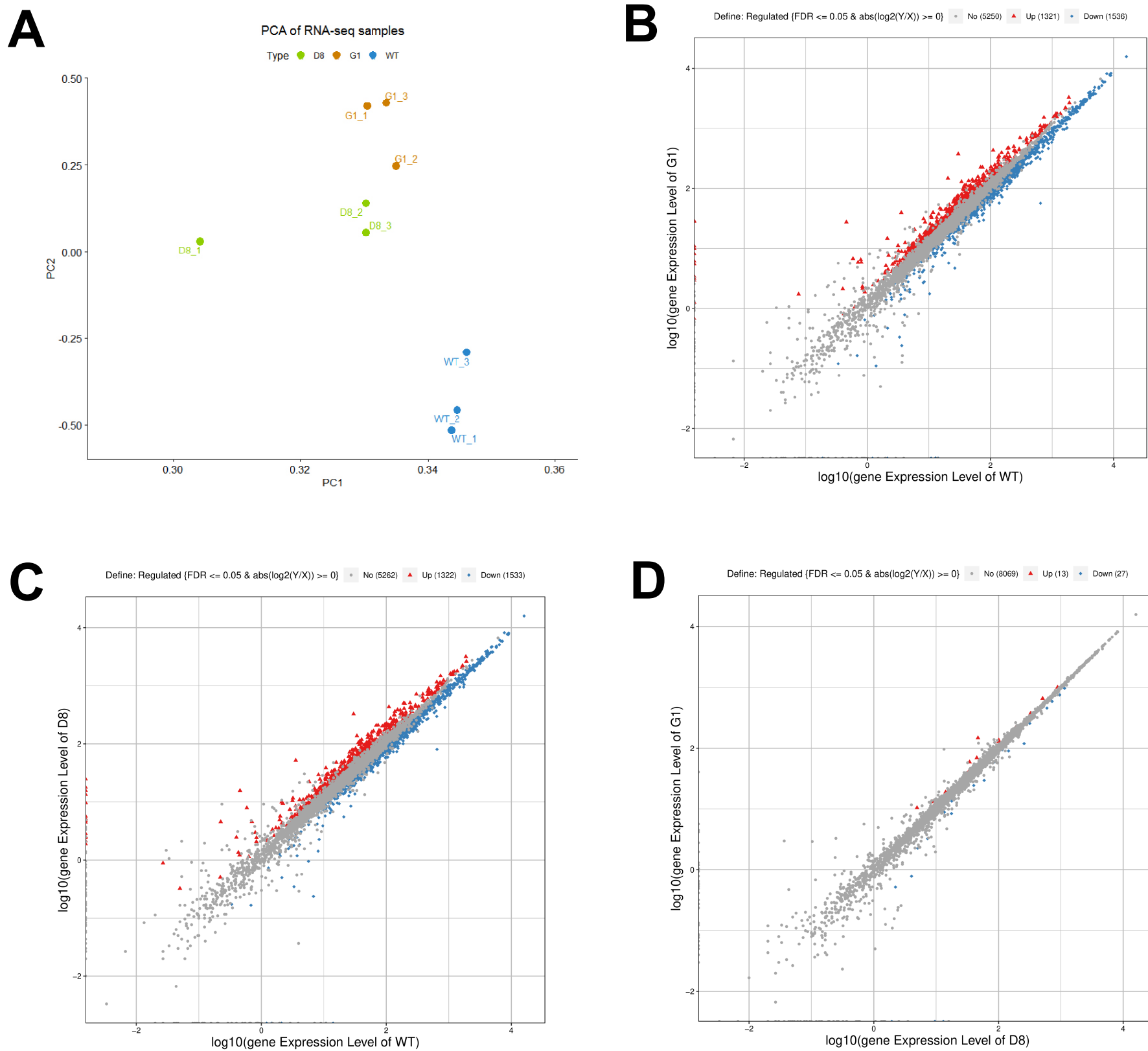

Supplement: Supplementary file 7 — Additional file 7: Supplementary Fig. S1. Comparison of RNA-seq data for WT and LpBBS2-deficient parasites. (A) Principal component analysis of RNA-seq samples from WT (blue), LpBBS2 mutant clone D8 (green), and clone G1 (beige). (B-D) Scatter plot analysis showing upregulated (red), downregulated (blue), and non-significantly different genes between WT and clone G1 (B), WT and clone D8 (C), or clone D8 and clone G1 (D). [file 13071_2025_6704_MOESM7_ESM.pdf]
