# Supplementary material for: BBSome deficiency in Lotmaria passim reveals divergent functions in trypanosomatid parasites
Source: Parasit Vectors. 2025 Feb 18;18:60. doi: 10.1186/s13071-025-06704-3 (PMC11837635; doi:10.1186/s13071-025-06704-3)
Supplement: Supplementary file 8 — Additional file 8: Supplementary Table S1. Normalized read counts of dynein heavy and light chain mRNAs in wild-type and LpBBS2 mutants. [file 13071_2025_6704_MOESM8_ESM.docx]

| Gene | Log2 fold change | Adjusted *P*-value | WT_1 | WT_2 | WT_3 | D8_1 | D8_2 | D8_3 |
| --- | --- | --- | --- | --- | --- | --- | --- | --- |
| Dynein heavy chain | 0.190670529 | 0.003065021 | 1476.723 | 1414.448 | 1435.998 | 1764.998 | 1646.982 | 1529.304 |
| Dynein light chain | 0.25046061 | 9.23708E-06 | 1545.993 | 1578.368 | 1602.009 | 1776.286 | 1894.122 | 1949.585 |
|  |  |  |  |  |  |  |  |  |
| Gene | Log2 fold change | Adjusted *P*-value | WT_1 | WT_2 | WT_3 | G1_1 | G1_2 | G1_3 |
| Dynein heavy chain | 0.310218335 | 2.59169E-06 | 1494.987 | 1433.25 | 1453.557 | 1949.186 | 1668.647 | 1814.946 |
| Dynein light chain | 0.251208148 | 6.86015E-05 | 1565.114 | 1599.349 | 1621.598 | 2047.353 | 1834 | 1814.946 |

Table S1 Normalized read counts of dynein heavy and light chain mRNAs in wild-type and *LpBBS2* mutants
